# Establishment of a stable transfection and gene targeting system in *Babesia divergens*

**DOI:** 10.3389/fcimb.2023.1278041

**Published:** 2023-12-13

**Authors:** Eliana F. G. Cubillos, Pavla Snebergerova, Sarka Borsodi, Dominika Reichensdorferova, Viktoriya Levytska, Masahito Asada, Daniel Sojka, Marie Jalovecka

**Affiliations:** ^1^ Faculty of Science, University of South Bohemia in Ceske Budejovice, Ceske Budejovice, Czechia; ^2^ Institute of Parasitology, Biology Centre of the Czech Academy of Sciences, Ceske Budejovice, Czechia; ^3^ National Research Center for Protozoan Diseases, Obihiro University of Agriculture and Veterinary Medicine, Hokkaido, Obihiro, Japan

**Keywords:** *Babesia divergens*, transfection system, erythrocytes pre-loading, GFP-expression, bidirectional promoter, gene targeting, *6-cys-e* gene knockout

## Abstract

*Babesia divergens* is an emerging tick-borne pathogen considered as the principal causative agent of bovine babesiosis in Europe with a notable zoonotic risk to human health. Despite its increasing impact, considerable gaps persist in our understanding of the molecular interactions between this parasite and its hosts. In this study, we address the current limitation of functional genomic tools in *B. divergens* and introduce a stable transfection system specific to this parasite. We define the parameters for a drug selection system *hdhfr*-WR99210 and evaluate different transfection protocols for highly efficient generation of transgenic parasites expressing GFP. We proved that plasmid delivery into bovine erythrocytes prior to their infection is the most optimal transfection approach for *B. divergens*, providing novel evidence of *Babesia* parasites’ ability to spontaneously uptake external DNA from erythrocytes cytoplasm. Furthermore, we validated the bidirectional and symmetrical activity of *ef-tgtp* promoter, enabling simultaneous expression of external genes. Lastly, we generated a *B. divergens* knockout line by targeting a *6-cys-e* gene locus. The observed dispensability of this gene in intraerythrocytic parasite development makes it a suitable recipient locus for further transgenic application. The platform for genetic manipulations presented herein serves as the initial step towards developing advanced functional genomic tools enabling the discovery of *B. divergens* molecules involved in host-vector-pathogen interactions.

## 1 Introduction

*Babesia divergens*, is an intraerythrocytic apicomplexan parasite transmitted by the tick *Ixodes ricinus* and is the primary cause of bovine babesiosis in Europe having a significant impact on the cattle industry (Zintl et al., 2003). Notably, human babesiosis caused by *B. divergens* is associated with severe and fatal outcomes of the disease, making it a zoonosis of concern in Europe (Hildebrandt et al., 2021). In comparison to other bovine infecting *Babesia* species, there is limited knowledge about the biology of *B. divergens*, and many questions concerning crucial processes of this parasite remain unanswered. Further investigation of mechanisms responsible for host cell invasion, intracellular parasitism, and parasite-host interactions, is required to improve our understanding of *B. divergens* and to develop innovative strategies aimed at preventing its transmission (Jalovecka et al., 2019).

The identification of factors responsible for the virulence and pathogenesis of *B. divergens* faces obstacles, primarily due to the absence of genetic manipulation tools necessary to study individual function of encoded proteins and enzymes. Transfection protocols and reverse genetics techniques are well established in several apicomplexans, including the primary model species *Plasmodium* and *Toxoplasma* (Limenitakis and Soldati-Favre, 2011; Vieira et al., 2021), but also in *Cryptosporidium* (Vinayak et al., 2015) and *Theileria* (De Goeyse et al., 2015). These tools are key to functionally characterize proteins and enzymes and determine them as potential targets for intervention. *Babesia*-specific transfection systems, both transient and stable, have been successfully developed for various *Babesia* species, including *Babesia bovis* (Suarez and McElwain, 2009; Suarez and McElwain, 2010), *Babesia ovata* (Hakimi et al., 2016), *Babesia bigemina* (Silva et al., 2016), *Babesia gibsoni* (Liu et al., 2018a), *Babesia ovis* (Rosa et al., 2019), *Babesia microti* (Jaijyan et al., 2020) and *Babesia duncani* (Wang et al., 2022). Such systems have proven to be vital tools for conducting comparative functional genomic analyses, providing valuable insights into the parasite’s biology. These insights include understanding host-pathogen interactions during the blood cycle (Asada et al., 2012a), as well as interactions between the parasite and tick vectors (Hussein et al., 2017). Therefore, there is an urgent need to develop a reliable platform for *B. divergens*-specific transgenic techniques to study key steps of this parasite’s life cycle.

The introduction of a transfection system into a new model species requires a number of prerequisites, including continuous *in vitro* culture, the availability of the parasite’s genome, a reliable drug selection system and a highly-efficient transfection protocol (Suarez et al., 2017). In *B. divergens*, the continuous *in vitro* cultivation in human (Lobo, 2005) and bovine erythrocytes (Malandrin et al., 2004) is well-established. The *B. divergens* genome has been sequenced (Cuesta et al., 2014) and recent advances in comparative genomic data are also available (González et al., 2019; Rezvani et al., 2022). This knowledge, in combination with the described transfection parameters, selection drugs and electroporation protocols of related *Babesia* species, provide a background for the development of genetic tools specific to *B. divergens*. Thus, in this work, we define the parameters for a stable and highly efficient *B. divergens*-specific transfection system as the initial step for the establishment of a wider range of functional genomic tools for this parasite.

## 2 Materials and methods

### 2.1 Parasite cultivation

*Babesia divergens* strain 2210A G2 was cultivated *in vitro* in the suspension of commercially available bovine erythrocytes (BioTrading) under constant conditions of 37°C and 5% CO_2_ by a previously described procedure (Jalovecka et al., 2018). The cultivation media was composed of RPMI 1640 (Lonza), amphotericin B (Merck, c=250µg/ml), gentamicin sulfate (Merck, c=10mg/ml) and supplemented with heat-inactivated fetal bovine serum (Capricorn) in 20% volume. Culture growth was monitored on thin blood smears stained by Diff-Quik (Siemens, Germany) under the BX53F light microscope (Olympus) at a magnification of 1000×.

### 2.2 Application of flow cytometry for parasitemia and GFP signal monitoring

To introduce a large-scale parasitemia monitoring method, we employed nuclear staining followed by a flow cytometry analysis. Infected red blood cells were pelleted, washed with 1× phosphate buffered saline (PBS), and fixed in a solution of 4% paraformaldehyde and 0.025% glutaraldehyde in PBS for 30 min at room temperature (RT). The fixed cells were subsequently washed twice with PBS (600×*g*, 3 min) and stored at 4°C for up to three weeks. To analyze parasitemia, the cells were stained with 0.02mM Ethidium Homodimer 1 (EthD-1, Biotinum) diluted in PBS for 30 min at RT. Afterwards, the stained samples were washed twice (600×*g*, 3 min) with PBS and analyzed using a FACS CantoII flow cytometer and Diva software provided by BD Biosciences, including the determination of Median Fluorescence Intensity (MFI) values for the GFP signal. When needed, flow cytometry was utilized to determine the GFP signal from transgenic parasites. Fixed parasites samples stained with EthD-1 were incubated with an Anti-GFP Polyclonal Antibody conjugated with Alexa Fluor™ 488 (Thermofisher), diluted 100× in PBS for 30 min. The samples were then washed twice with PBS (600×*g*, 3 min) and analyzed in the same manner as stated above.

### 2.3 Evaluation of the inhibitory effect of selection drugs

The inhibitory effect of two standard selection drugs, WR99210 (selection marker *hdhfr - human dihydrofolate reductase -* gene, kindly provided by Jacobus Pharmaceutical) and blasticidin S (BSD, selection marker *blasticidin S deaminase* gene, Merck) was tested in *B. divergens in vitro* system. Inhibitory assays were conducted in 96-well plate format in biological triplicates for 8 consecutive days. Media supplemented either with WR99210 (concentration ranging from 20 nM to 0.019 nM) or BSD (concentration ranging from 16 µg/ml to 0.0156 µg/ml) were exchanged in an interval of 48 hours, DMSO diluted in cultivation media served as solvent control. Culture samples were collected at two-day intervals, fixed, and analyzed using flow cytometry as stated in chapter 2.1.

### 2.4 Plasmids construction

To construct *B. divergens*-specific plasmids for both episomal and intragenomic *gfp* expression, we first identified, PCR amplified, and sequenced *B. divergens*-specific untranslated regions (UTRs): the 5’ UTR of the *actin* gene (Bdiv_007890; length: 2002 bp), the 5’ UTR of the *elongation factor tu gtp binding domain family protein* (*ef-tgtp*) gene (Bdiv_030590; length: 696 bp), and the 3’ UTR of the *chloroquine resistance transporter* (CRT) gene (Bdiv_036760; length: 1496 bp). The coding sequences of *gfp, hdhfr*, and *bsd* genes were obtained from *B. bovis*-specific plasmids (Asada et al., 2015). The target locus for intragenomic insertion of the GFP-expressing cassette, the *B. divergens 6-cys-e* gene (6-cysteine (E), Bdiv_004560c), and its 5’ and 3’ UTRs, were PCR amplified and sequenced. The final plasmid included two homology regions (HRs): HR1 (length: 817 bp) spanning the 5’ UTR and the beginning of the gene CDS, and HR2 (length: 802 bp) spanning the end of the gene CDS and the 3’ UTR to allow homologous recombination.

The plasmids were assembled in several subsequent steps using NEBuilder® HiFi DNA Assembly Kit (New England BioLabs) following the manufacturer’s protocols. The procedure involved the linearization of the plasmid’s backbone using specific restriction enzymes and PCR amplification of the desired loci. Detailed information about individual plasmid parts and restriction sites are depicted in plasmid maps (**Supplementary Data**; https://doi.org/10.6084/m9.figshare.24433384.v1). After assembly, the plasmids were transformed into NEB 5-alpha Competent *E. coli* cells (New England Biolabs) and grown on LB agar plates supplemented with ampicillin, following the manufacturer’s protocols. Individual colonies obtained from the plates were grown in LB medium supplemented with ampicillin for 12-16 hours, and the plasmid DNA was extracted using the NucleoSpin® Plasmid kit (Macherey-Nagel) and verified by sequencing. To isolate and purify plasmid DNA on a large scale for transfections, the NucleoBond® Xtra Midi kit (Macherey-Nagel) was employed.

The plasmids designed for episomal GFP expression were transfected in circular form, as detailed in the subsequent chapter. The plasmid intended for intragenomic *gfp* expression was linearized using the FseI restriction enzyme before the transfection.

### 2.5 Transfection

Three different protocols were employed for the transfection of *B. divergens* parasites: electroporation of infected RBCs (iRBCs), electroporation of free merozoites, and electroporation of uninfected bovine RBCs (uRBCs). All transfection protocols included a “plasmid solution”: 10 µg of the plasmid that was precipitated in ethanol, resuspended in 20 µl of filtered milliQ water, and mixed with 100 µl of nucleofector solution from Basic Parasite Nucleofector® Kit 2 (Lonza). Transfections were performed in a device ‘Amaxa’ Nucleofector 2b (Lonza) and an electric pulse using the V-024 program was applied to the mixture of plasmid solution and iRBCs/free merozoites/uRBCs solution. Transfected samples were then transferred into a mixture of 2 ml cultivation media and 100 µl of uRBCs/uRBCs/iRBCs, respectively. Twenty-four hours post-transfection, the medium was replaced, and the 5 nM WR99210 selection drug was added. The medium containing the drug was changed daily during the experiments.

Transfection of *B. divergens* iRBCs was adapted from the previously described protocol for *B. bovis* (Suarez et al., 2007; Asada et al., 2012b): 100 µl of iRBCs with ~15% parasitemia were washed in pre-warmed PBS and mixed with the plasmid solution. Transfection of free merozoites included 100 μl of iRBCs with ~15% parasitemia that were washed in pre-warmed PBS and filtered twice with a 1.2 µm Acrodisc® syringe filter unit (Pall corporation) as described in (Hakimi et al., 2021a). The filtered suspension was then centrifuged (600×*g*, 3 min) to remove the residues of RBCs, and free merozoites resuspended in 100 μl of PBS were mixed with the plasmid solution. The transfection of uRBCs (also referred to as DNA or plasmid pre-loading of RBCs) was adapted from *P. falciparum* protocol (Deitsch et al., 2001). Briefly, 100 μl of uRBCs were washed with PBS and mixed with the plasmid solution. After the transfection, the mixture of DNA-loaded uRBCs was mixed with 2 ml cultivation media supplemented with 100 µl of iRBCs. Three independent experiments were conducted to evaluate the efficiency of the above presented different transfection protocols.

### 2.6 Limiting dilution/cloning

To obtain clonal populations with correctly integrated plasmid, the parasites transfected with the linearized plasmid were subjected to serial dilutions: 0.3 parasites were seeded per well in a 96-well plate and grown for 10 days with medium exchanges every second day. Obtained clones were expanded for DNA extraction and the correct integration of the plasmid was confirmed by PCR using the primers listed in **Supplementary Table 1**.

### 2.7 Growth curve analyses

To analyze the growth pattern of GFP-expressing and knockout (*6-cys-e* gene disruption) parasites, their intraerythrocytic development was monitored for 96 hours and compared to a wild-type parasite lineage. All experiments were conducted in biological triplicates, and parasitemia was determined every 12 hours through blood smears and flow cytometry.

### 2.8 DNA extraction and PCR

*B. divergens* gDNA was isolated from iRBCs (~10% parasitemia) using the NucleoSpin® Blood DNA isolation kit (Macherey-Nagel) according to the manufacturer’s instructions and stored at -20°C. PCR reactions were performed in the T100 Thermal Cycler (BioRad) following the protocol for Q5® High-Fidelity 2X Master Mix (New England BioLabs) and visualized on agarose gel using Gel logic 112 transilluminator (Merck).

### 2.9 RNA extraction, reverse transcription, and qPCR

Total RNA from *B. divergens in vitro* cultures was extracted using TRI Reagent® (Merck) followed by chloroform phase separation and isolation with the NucleoSpinRNA II kit (Macherey-Nagel). To eliminate gDNA residues, the RNA samples were treated with the TURBO DNA-free™ Kit (Invitrogen) and stored at -80°C. Reverse transcription was performed with 1 μg of RNA using the Transcriptor First Strand cDNA Synthesis Kit (Roche), following the manufacturer’s protocol, and stored at -20°C. Transcript levels of *gfp* and *hdhfr* genes were quantified in biological as well as technical triplicates using LightCycler® 480 SYBR Green I Master (Roche) with specific primers for each transcript (**Supplementary Table 1**) in CFX96 Touch Real-Time PCR Detection System (BioRad). The relative *gfp* and *hdhfr* genes expression was normalized to the *B. divergens gapdh* gene (Bdiv_010720) using the ΔΔCt method (Jalovecka et al., 2016).

### 2.10 Fluorescence microscopy

GFP-expressing *B. divergens in vitro* cultures were smeared, air-dried and fixed with 4% paraformaldehyde and 0.075% glutaraldehyde in PBS for 30 min at RT on Superfrost Plus™ Adhesion Microscope Slides (Thermo Fisher Scientific). After fixation, the samples were washed with PBS (3 × 5 min) and incubated with 0.01% Triton X-100 (Merck) diluted PBS for 30 min at RT to permeabilize cell membranes. Anti-GFP Polyclonal Antibody conjugated with Alexa Fluor™ 488 (Thermofisher) was diluted 100× in 0.01% Triton X-100 and applied to samples for 1 hour at RT. Subsequently, the samples were washed in PBS (3 × 5 min) and stained with 300 nM DAPI diluted in PBS for 10 min at RT. After another round of washing with PBS (3 × 5 minutes), the samples were mounted in DABCO (Merck). GFP signal was examined by BX53F fluorescence microscope (Olympus) and processed in Fiji (ImageJ) software.

### 2.11 Statistics

The statistical analyses, IC50 calculations, and graphs were created in GraphPad Prism software (version 7.0a). The inhibitory effect of selection drugs was evaluated by one-way analysis of variance (ANOVA), Kolmogorov-Smirnov test, and the Bartlett test passed. The expression of *gfp* and *hdhfr* genes was compared with the non-parametric Mann-Whitney test. * = p < 0.05, ** = p < 0.01, *** = p < 0.001.

## 3 Results

### 3.1 *B. divergens* is inhibited by WR99210 and blasticidin S

**Figure 1:**
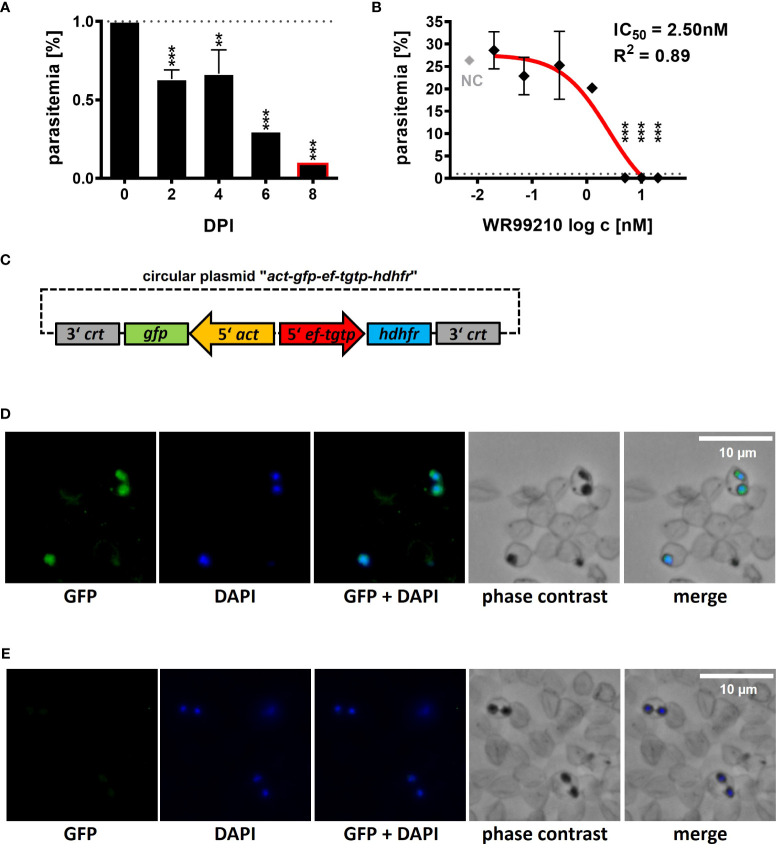
Introduction of a *B*. *divergens* GFP-expressing reporter line. **(A)** Inhibition of *B*. *divergens in vitro* growth in bovine RBCs by 5nM WR99210. **(B)** Determination of IC_50_ value for WR99210 using non-linear regression with a dose-response curve and regression factor (R^2^) based on *B*. *divergens* parasitemia levels on 8 DPI. Individual concentrations were transformed (log c) prior to analysis. The result represents the mean of three independent replicates, with error bars indicating standard deviations. The grey dotted line represents the initial parasitemia (1%). NC, non-treated culture; IC50, half-maximal inhibitory concentration; DPI, days post infection; **,p < 0.01; ***,p < 0.001. One-way ANOVA was performed for statistical analysis. **(C)** Circular plasmid *act-gfp-ef-tgtp-hdhfr* used for the introduction of a GFP-expressing *B*. *divergens* reporter lineage from an extrachromosomal replicating episome. *crt*, chloroquine resistance transporter; *gfp*, green fluorescent protein; *act*, actin; *ef-tgtp*, Elongation Factor Tu GTP binding domain family protein; *hdhfr*, human dihydrofolate reductase. Detailed map is included in **Supplementary Data** and available online https://doi.org/10.6084/m9.figshare.24433384.v1. **(D)** Immunofluorescence microscopy of episomally GFP-expressing parasites selected with WR99210. **(E)** Immunofluorescence microscopy of wild-type parasites. Fixed thin blood smears were stained with Anti-GFP Polyclonal Antibody conjugated with Alexa Fluor™ 488, and nuclei were stained with DAPI. Dotted red circles indicate RBCs.

Since the establishment of stable transfection system requires a reliable drug selection, we conducted tests to assess the impact of two commonly used selection drugs, WR99210 and BSD, on the growth of *B. divergens*. Both drugs demonstrated inhibitory activity against *B. divergens* in our *in vitro* system. WR99210 was found to be effective at a concentration of 5 nM (**Figure 1A**), while BSD exhibited inhibitory effects at a concentration of 4 μg/ml (equivalent to 8.72 μM) (**Supplementary Figure 1A**).

At 5 nM, WR99210 significantly inhibited parasite growth by 2 days post infection (DPI), reducing parasitemia to 0.1% by 8 DPI compared to the initial 1% (0 DPI, **Figure 1A**), whereas the non-treated culture (NC) grew up to a parasitemia of 25.35 ± 2.33% on 8 DPI (**Figure 1B**). Inhibitory effects were also observed at 10 and 20 nM concentrations, while concentrations of 1.25, 0.31, 0.07, and 0.02 nM did not exhibit significant growth inhibition. The IC_50_ of WR99210 in *B. divergens* bovine RBCs, determined from parasitemia values measured on 8 DPI, was calculated 2.50 nM with an R^2^ value of 0.89 (**Figure 1B**).

BSD at 4 μg/ml (8.72 μM) significantly decreased *B. divergens* growth: on 8 DPI, parasitemia decreased to 0.4% ± 0.1 from an initial 1% (0 DPI, **Supplementary Figure 1A**), while the NC group showed 23.85% ± 2.48% parasitemia (**Supplementary Figure 1B**). BSD also exhibited inhibitory effects at concentrations of 8 and 16 μg/ml (17.43 and 34.86 μM, respectively), but no impact on growth was observed with concentrations of 1, 0.25, 0.0625, and 0.015 μg/ml (equivalent to 2.18, 0.54, 0.14 and 0.03 μM, respectively). The IC_50_ of BSD in *B. divergens in vitro* system was calculated as 2.61 μg/ml (5.69 μM), with an R^2^ value of 0.90, based on parasitemia values measured on 8 DPI (**Supplementary Figure 1B**).

### 3.2 Introduction of a *B. divergens* GFP-expressing reporter line

With a reliable selection system, we further proceeded to develop a *B. divergens* GFP-expressing reporter line. We designed and *de novo* constructed a circular plasmid “*act-gfp-ef-tgtp-hdhfr*” containing the GFP reporter cassette, where the *gfp* gene expression was driven by the *actin* gene promoter. The *hdhfr* gene, conferring resistance to the drug WR99210, was under the control of the promoter of the Elongation Factor Tu GTP binding domain family protein (*ef-tgtp*) (**Figure 1C**; **Supplementary Data**; https://doi.org/10.6084/m9.figshare.24433384.v1). The plasmid was delivered into *B. divergens*-infected bovine RBCs, and WR99210-resistant parasites emerged after 12 to 14 days post transfection (DPT). By 16 to 18 DPT, parasitemia reached 5%. Throughout this study, the GFP fluorescence signal was consistently detected (**Figure 1D**) under WR99210 pressure. No GFP signal was detected in wild-type parasites (**Figure 1E**).

Similarly, the BSD-resistant parasites appeared between 12 to 15 DPT after delivery of the modified plasmid in which the same promoter was used to drive the expression of the *blasticidin S deaminase* gene as in the above-mentioned validated *hdhfr*-WR99210 selection system. However, despite several attempts to stabilize the culture, the parasitemia remained low, and the parasites were no longer detectable after 24 DPT (data not shown). Due to the low parasitemia, the GFP signal could not be adequately confirmed. Consequently, in the following experiments, the *hdhfr*-WR99210 selection system was used.

### 3.3 *B. divergens* spontaneously uptakes plasmid DNA from bovine RBCs cytoplasm

**Figure 2:**
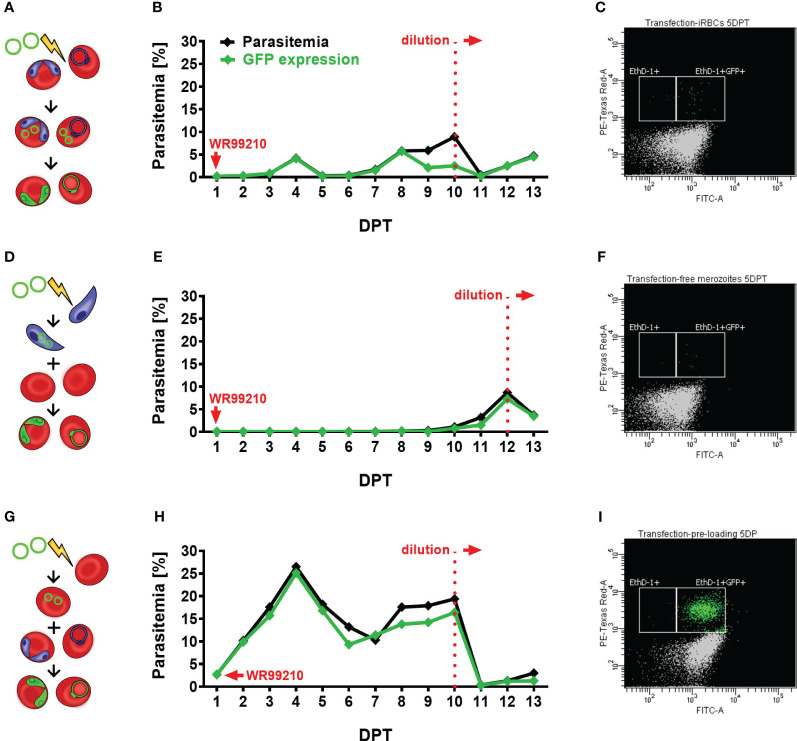
Assessment of different transfection protocols in *B. divergens in vitro* system. **(A–C)** Direct transfection of iRBCs. **(A)** Schematic representation of the transfection protocol, **(B)** parasitemia and GFP fluorescence signal monitoring in transfected parasite culture, **(C)** parasitemia of GFP-expressed parasites on 5 DPT. **(D–F)** Electroporation of free merozoites: **(D)** Schematic representation of the transfection protocol, **(E)** parasitemia and GFP fluorescence signal monitoring in transfected parasite culture, **(F)** parasitemia of GFP-expressed parasites on 5 DPT. **(G–I)** Plasmid delivery into uRBCs prior to their infection: **(G)** Schematic representation of the transfection protocol, **(H)** parasitemia and GFP fluorescence signal monitoring in transfected parasite culture, **(I)** parasitemia of GFP-expressed parasites on 5 DPT. In all experiments, parasite growth and GFP expression were evaluated using flow cytometry. Culture samples were fixed on daily intervals, stained with EthD-1 to determine total parasitemia, and labeled with an Anti-GFP Polyclonal Antibody conjugated with Alexa Fluor™ 488. The graphs represent the results of a single experiment, the observed outcome has been confirmed by two additional independent experiments. iRBCs, infected red blood cells; uRBCs, uninfected RBCs; GFP, green fluorescent protein; EthD-1, Ethidium Homodimer 1; DPT, days post transfection.

Since the GFP-*hdhfr* expression plasmid was verified, we proceeded to optimize the transfection protocol, with the aim of establishing a versatile platform for *B. divergens*-specific transgenic techniques. To achieve this, we explored three different methods for delivering the circular plasmid “*act-gfp-ef-tgtp-hdhfr*” into parasite cells: the widely used and well-established direct iRBCs transfection (**Figure 2A**) (Suarez and McElwain, 2010; Asada et al., 2012b), the less common but validated method of electroporation of *Babesia* free merozoites (**Figure 2D**) (Suarez and McElwain, 2008), and lastly, the plasmid delivery into uRBCs before their infection (**Figure 2G**), followed by their coculturing with wild-type (WT) parasites. The latter method of erythrocytes pre-loading has not been previously validated in any *Babesia*-transgenic system, but in *P. falciparum*, it has demonstrated higher efficiency when compared to other methods (Deitsch et al., 2001; Skinner-Adams et al., 2003; Hasenkamp et al., 2012).

After comparing the three transfection methods (**Figure 2**), the plasmid pre-loading into uRBCs proved to be the most efficient transfection protocol in *B. divergens in vitro* system (**Figures 2H, I**) in all three independent experiments. The observed GFP signal from parasites confirmed the spontaneous uptake of plasmid DNA from the bovine host cell cytoplasm into *B. divergens* intraerythrocytic stages. Additionally, parasitemia of nearly 3% of GFP-expressing parasites was detected 1 DPT (**Supplementary Figure 2C**) and gradually increased up to 17%. However, the parasites showed signs of stress due to overgrowth, likely associated with the decreased ratio of GFP expression to total parasitemia observed from 8 DPT. Thus, the cultures were diluted on 10 DPT, when the MFI of GFP signal was measured as 1411, to avoid stressful conditions that could interfere with parasite growth. Following dilution, the parasites quickly re-emerged with MFI value on 11DPT 1419, and parasitemia exceeded 3% by 13 DPT. Throughout the experiment, when comparing total parasitemia with the number of GFP-expressing parasites, the same trend of growth was observed. This confirms the successful transfection and plasmid maintenance by the parasites. Moreover, this technique demonstrated the highest viability compared to the other tested methods, as evidenced by a blood smear taken on 1 DPT (**Supplementary Figure 2F**). After the direct electroporation of iRBCs, a harmful effect on parasites was observed, as evidenced by the presence of multiple dead parasites on 1 DPT and a high rate of RBC lysis (**Supplementary Figures 2A, D**). Despite this, the GFP-expressing parasite population gradually increased over time, reaching over 5% parasitemia by 8 DPT, but remained at lower levels compared to the pre-loaded transfectants (**Figures 2D, E**). Signs of overgrowth were detected from 9 DPT, necessitating dilution of the culture on 10 DPT, when MFI reached 626. Following dilution, the parasite population re-emerged with detected MFI 826 on 11 DPT and reached a stable parasitemia with sustained GFP expression. In contrast, the electroporation of *B. divergens* free merozoites displayed delayed appearance of transgenic parasites (**Figures 2F, G**). Despite a few viable parasites being detected on 1 DPT (**Supplementary Figures 2B, E**), the first GFP-expressing parasites only emerged around 10 DPT. This delay may be attributed to the fragility of the free merozoite population, resulting in reduced merozoite viability after electroporation, or a low efficiency of the transfection protocol, or a combination of both factors. By 12 DPT, the parasitemia of transgenic parasites exceeded 7% with MFI value 967, and the observed majority of GFP-expressing parasites indicates successful transformation. We then proceeded with culture dilution to observe whether parasites would be able to re-emerge. Indeed, after the dilution, GFP-expressing parasites exponentially multiplied with MFI value 1726 detected on 13 DPT, indicating the continuous growth of a successfully transfected population.

### 3.4 Validation of novel bidirectional promoter with symmetrical activity

**Figure 3:**
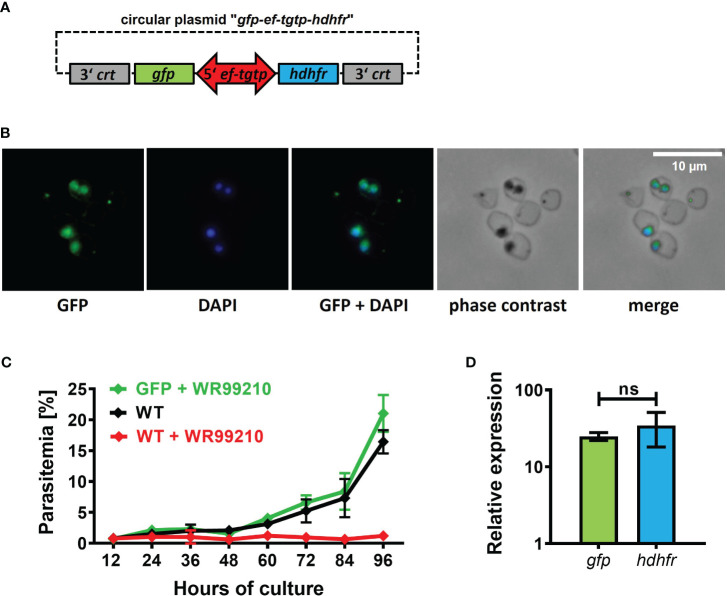
Validation of novel bidirectional promoter with symmetrical activity. **(A)** Circular plasmid *gfp-eftgtp-hdhfr* employing bidirectional promoter of the *elongation factor tu gtp binding domain family protein* (*eftgtp*) gene. *crt*, chloroquine resistance transporter; *gfp*, green fluorescent protein; *hdhfr*, human dihydrofolate reductase. Detailed map is included in **Supplementary Data** and available online https://doi.org/10.6084/m9.figshare.24433384.v1. **(B)** Episomally GFP-expressing parasites selected with WR99210. Fixed thin blood smears were stained with an Anti-GFP Polyclonal Antibody conjugated with Alexa Fluor™ 488, and nuclei were counterstained with DAPI. Dotted red circles indicate RBCs. **(C)** The growth curve of GFP-expressing parasites under WR99210 selection pressure was compared to WT non-treated parasites and WR99210 treated. Stained blood smears were employed for growth monitoring. WT, wild-type **(D)** The relative quantification of *gfp* and *dhfr* gene transcripts driven by the *eftgtp* bidirectional promoter. Non-parametric Mann-Whitney test was performed for statistical analysis. ns, not significant.

In most *Babesia* species, the promoters controlling the expression of *elongation factor-1 alpha* (*ef1-α*) exhibit robust, constitutive, and bidirectional activity in transgenic applications (Liu et al., 2018b). Consequently, when introducing transgenic applications into our *B. divergens* system, we selected the promoter of Bdiv_030590, a gene annotated in PiroplasmaDB as “*the translation elongation factor EF-1, subunit alpha protein, putative*”. However, a more comprehensive *in silico* analysis of the Bdiv_030590 gene revealed distinct differences from the typical structure of well characterized *ef1-α* promoters in other *Babesia* species (Suarez et al., 2006; Silva et al., 2016; Liu et al., 2018b; Wang et al., 2022). Instead, the arrangement of Bdiv_030590 gene closely resembles the locus of syntenic gene BBOV_II000640, which codes for Elongation factor Tu GTP binding domain family protein (Dr. Carlos Suarez, personal communication). Therefore, we designated the promoter of Bdiv_030590 gene as the “*ef-tgtp*”. Based on its position between two genes (Bdiv_030590 and Bdiv_030580c) in opposite direction, we hypothesized that it might possess bidirectional activity, allowing us to reduce the size of the original plasmid “*act-gfp-ef-tgtp-hdhfr*” to enhance its versatility. By removing the actin promoter sequence from the original plasmid, we created a circular plasmid “*gfp-ef-tgtp-hdhfr*” which contained both the GFP reporter cassette and the *hdhfr* gene-based resistance cassette under the control of the *ef-tgtp* promoter in a head-to-head orientation (**Figure 3A**; **Supplementary Data**; https://doi.org/10.6084/m9.figshare.24433384.v1). The stable GFP expression in episomally transfected parasites and their continuous growth under drug selection pressure confirmed a bidirectional activity of the *ef-tgtp* promoter (**Figures 3B, C**). Moreover, the promoter activity was symmetrical, as both transcripts of the *gfp* and *hdhfr* genes exhibited expression levels with no significant difference (**Figure 3D**), which makes this promoter a widely adaptable tool for other transgenic applications.

### 3.5 Targeted *B. divergens* intragenomic insertion shows dispensability of the *6-cys-e* gene

**Figure 4:**
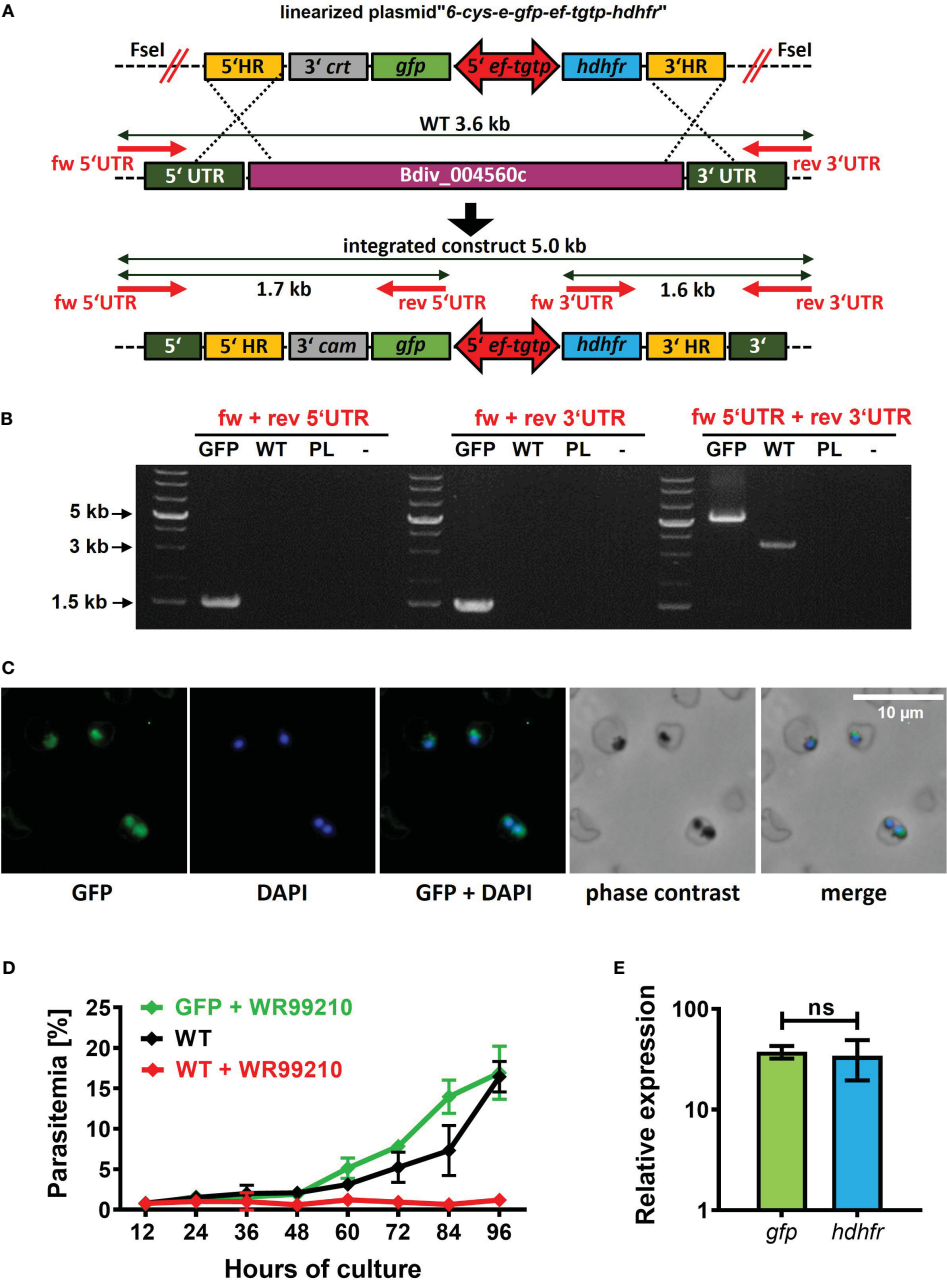
Targeted *B*. *divergens* intragenomic insertion. **(A)** Intragenomic insertion of GFP reporter cassette into *B*. *divergens 6-cys-e g*ene (Bdiv_004560c) using linearized plasmid “*6-cys-e-gfp-ef-tgtp-hdhfr.*” HR, homology regions; *crt*, chloroquine resistance transporter; *gfp*, green fluorescent protein; *ef-tgtp*, Elongation Factor Tu GTP binding domain family protein; *hdhfr*, human dihydrofolate reductase; UTR, untranslated regions; WT, wild-type; red arrows represent positions of primers used for integration test. Detailed map is included in **Supplementary Data** and available online https://doi.org/10.6084/m9.figshare.24433384.v1. **(B)** Integration test demonstrating the correct integration of both the GFP reporter cassette and the *hdhfr* gene-based resistance cassette into desired locus. PL, plasmid. **(C)** Immunofluorescence microscopy capturing GFP expression in clonal transgenic parasites. Fixed thin blood smears were stained with an Anti-GFP Polyclonal Antibody conjugated with Alexa Fluor™ 488, and nuclei were counterstained with DAPI. Dotted red circles indicate RBCs. **(D)** The growth curve of clonal transgenic parasites under WR99210 selection pressure was compared to wild-type (WT) non-treated parasites and WR99210 treated. Growth curves were determined through blood smears. **(E)** The relative quantification of *gfp* and *dhdfr* gene transcripts driven by the *ef-tgtp* bidirectional promoter. Non-parametric Mann-Whitney test was performed for statistical analysis. ns, not significant.

To complete the development of a *B. divergens*-specific transgenic platform, we proceeded with the direct insertion of a foreign gene into the parasite genome. In already established *Babesia*-transgenic systems, this process is commonly achieved through homologous recombination mechanisms (Suarez et al., 2017). To target a dispensable locus/gene for parasite blood stages, we chose the *B. divergens 6-cys-e* gene (Bdiv_004560c). The ortholog of this gene in *B. bovis* has been demonstrated to be dispensable for the intraerythrocytic life cycle of this parasite. To introduce the GFP reporter cassette into the selected locus, we designed the plasmid “*6-cys-e-gfp-ef-tgtp-hdhfr*” (**Supplementary Data**; https://doi.org/10.6084/m9.figshare.24433384.v1). This plasmid contains two homology regions (HRs) that are homologous to the 5’ and 3’ UTRs of *6-cys-e* gene. Within these HRs, the GFP reporter cassette and the *hdhfr* gene-based resistance cassette were positioned in a head-to-head orientation, separated by the *ef-tgtp* promoter (**Figure 4A**). The pre-loading of the linearized plasmid into bovine uRBCs resulted in the emergence of WR99210-resistant parasites on 12 DPT that gradually increased, surpassing 5% parasitemia by 15 DPT. Following clonal selection, the successful intragenomic insertion was confirmed (**Figure 4B**), and GFP signal was detected (**Figure 4C**). Furthermore, the symmetrical activity of *ef-tgtp* bidirectional promoter was reconfirmed (**Figure 4E**). The continuous growth of the obtained clonal population under the drug pressure with no significant differences (**Figure 4D**) confirms that the expression of the *6-cys-e* gene is not essential for *B. divergens* intraerythrocytic multiplication. The dispensability of the *6-cys-e* gene makes it a suitable recipient locus for more advanced *B. divergens*-specific transgenic systems requiring target integration of the parasite genome. However, the inserted sequences should be driven by external and constitutive promoters to assure stable expression of the transgenes.

## 4 Discussion

In this study, we introduce a stable and highly efficient transfection protocol for *B. divergens*, an economically and medically important European tick-transmitted apicomplexan parasite. We established *B. divergens*-specific platform for genetic manipulations within the bovine RBCs cultivation system with the goal of advancing the development of functional genomic tools for this parasite.

The establishment of a reliable selection system is the primary step in developing transgenic techniques for novel model species. In *Babesia* species, the *human dihydrofolate reductase* (*hdhfr*) gene, which provides resistance to the drug WR99210 (Gaffar et al., 2004), serves as the widely used selectable marker. We confirmed *B. divergens*’s susceptibility to WR99210, resulting in complete growth inhibition at a concentration of 5nM, which corresponds to the standard dose in *Babesia* transgenic systems (e.g. (Asada et al., 2012b; Hakimi et al., 2016; Wang et al., 2022). Consequently, we employed this dose for successful selection of GFP-expressing *B. divergens* transgenic populations (**Figure 1**). While *B. divergens* displayed sensitivity to Blasticidin S (BSD) (**Supplementary Figure 1**), another established selection drug in *Babesia* transgenic systems (Suarez and McElwain, 2010), our attempts to select and maintain transgenic parasites using a 4 μg/ml (8.72 μM) concentration were unsuccessful. A recent pre-print (Elsworth et al., 2023) demonstrated successful selection of transgenic *B. divergens* using 20 μg/ml (43.58 μM) of BSD, suggesting the need for higher concentrations. However, BSD selection varies widely in effectiveness among different *Babesia* species, ranging from 2 μg/ml (4.36 μM) in *B. bovis* (Suarez and McElwain, 2009) to no inhibitory effects, even at 100 μg/ml (217.91 μM) in *B. duncani* (Wang et al., 2022). This indicates that BSD selection may not be universally applicable, necessitating further investigations to confirm its suitability for individual *Babesia* species.

*Babesia*-specific transfection protocols typically involve electroporation of infected RBCs (iRCBs) or, less commonly, transfection of free (purified) merozoites (Hakimi et al., 2021b). While the alternative approach based on vector DNA electroporation of uninfected RBCs (uRBCs) cells has proven highly effective in *P. falciparum* (Deitsch et al., 2001; Skinner-Adams et al., 2003; Hasenkamp et al., 2012), its application in *Babesia* species is still unverified. In this study, we evaluated all three listed methods and found that each produced transgenic GFP-expressing *B. divergens* parasites. Notably, the ‘pre-loading’ of uRBCs method resulted in higher parasitemia compared to electroporation of iRBCs or purified merozoites (**Figure 2**), consistent with observations in *P. falciparum* (Skinner-Adams et al., 2003; Hasenkamp et al., 2012). We hypothesize that this observation may be attributed to the absence of direct exposure of parasites to the intense electrical currents experienced during electroporation of iRCBs or free merozoites. Previous attempts to establish a *B. ovata* transfection protocol using the ‘pre-loading of uRBCs’ method were unsuccessful (Hakimi et al., 2016), possibly due to voltage and capacitance settings variations between the BioRad and the herein used Lonza (Amaxa) electroporation systems. Overall, the ‘pre-loading of uRBCs appears to be a most promising method for advancing genetic tools in *B. divergens*. Additionally, our findings illuminate the ability of *Babesia* parasites to spontaneously uptake external DNA from RBC cytoplasm, shedding new light on their intraerythrocytic development linked to disease.

The availability of *Babesia* genomes and the PiroplasmaDB database (Amos et al., 2022) have facilitated the identification of regulatory regions controlling gene expression. The promoters controlling the expression of *elongation factor-1 alpha* (*ef1-α*) belong to the most used regulatory regions in transgenic applications of other *Babesia* species (Liu et al., 2018b). Therefore, we selected the promoter of Bdiv_030590 gene, referred to as *B. divergens ef1-α, putative* in PiroplasmaDB for our experiments. However, the arrangement of Bdiv_030590 gene locus mirrors the structure of the syntenic gene locus coding for *B. bovis* Elongation Factor Tu GTP binding domain family protein, rather than the validated canonical *ef1-α* promoters found in other *Babesia* species (Suarez et al., 2006; Silva et al., 2016; Liu et al., 2018b; Wang et al., 2022) (Dr. Carlos Suarez, personal communication). Thus, we designated the promoter employed in our study as the “*ef-tgtp*” and experimentally validated its ability to control external gene expression. Notably, we confirmed its constitutive bidirectional activity as the *ef-tgtp* promoter exhibited symmetrical activity in controlling both GFP reporter and the *hdhfr*-WR99210 resistance cassettes. This novel promoter has demonstrated its versatility in regulating external gene expression for various *B. divergens*-specific transgenic applications (**Figure 3**). The observed elevated GFP expression can be attributed to a combination of factors, including the use of the *eg-tgtp* bi-directional promoter, drug pressure, and episomal gene transcription. While our study did not identify adverse effects resulting from GFP overexpression on parasite viability, it is important to acknowledge that potential issues may arise with other proteins. Addressing such concerns would necessitate further optimization, considering all relevant expression parameters, with particular attention to the concurrent use of the same promoter for drug resistance and protein expression.

In this study, we aimed to establish a stable transfection system for *B. divergens*, which necessitates continuous expression of the gene of interest. This can be achieved through extrachromosomal replicating episomes or nuclear genome integration (Limenitakis and Soldati-Favre, 2011). To achieve this, we first successfully introduced transgenic *B. divergens* parasites expressing GFP from a circular plasmid, as illustrated in **Figures 1**–**3**. Subsequently, we generated clonal transgenic parasites exhibiting a GFP fluorescence signal, as demonstrated in **Figure 4**. These parasites had the original *6-cys-e gene* (Bdiv_004560c) locus replaced by both the GFP reporter and *hdhfr*-WR99210 resistance cassettes, driven by an external *ef-tgtp* promoter. This marks the first gene knockout in this species. Similar to observations in *B. bovis* (Alzan et al., 2017), the knockout of the *B. divergens 6-cys-e* gene displayed the same growth phenotype as the wild-type control, confirming the suitability of the locus for hosting transgenic cassettes. While exploring the role of the resulting 6-cys-E protein in sexual stages (Alzan et al., 2019), its necessity for completing the *B. divergens* life cycle within the *I. ricinus* tick vector was beyond this study’s scope, but the created lines serve as a valuable tool for such experiments that may lead to innovative transmission-blocking applications for *Babesia*.

In conclusion, we have established a highly efficient *B. divergens*-specific transfection protocol using plasmid delivery into uRBCs before infection and a reliable *hdhfr*-WR99210 drug selection system. We validated the bidirectional and symmetrical activity of the *ef-tgtp* promoter for the regulation of external gene expression. Additionally, we introduced *B. divergens* fluorescent reporter lineages through two distinct methods: extrachromosomal replicating episomes and nuclear genome targeting. Last but not least, we successfully generated a *B. divergens* 6-cys-e gene knockout line, confirming its non-essential role in blood stage replication. This transfection system has significant potential for advancing functional genomic tools in *B. divergens*, facilitating a deeper exploration of the crucial processes that govern parasitism within vertebrate host cells and transmission by tick vectors.

## Data availability statement

The original contributions presented in the study are included in the article/**Supplementary Material**, further inquiries can be directed to the corresponding author/s.

## Ethics statement

Ethical approval was not required for the studies on animals in accordance with the local legislation and institutional requirements because only commercially available established cell lines were used.

## Author contributions

EC: Conceptualization, Data curation, Formal Analysis, Investigation, Methodology, Writing – original draft, Writing – review & editing. PS: Data curation, Formal Analysis, Investigation, Methodology, Writing – review & editing, Funding acquisition. SB: Investigation, Methodology, Writing – review & editing. DR: Investigation, Methodology, Writing – review & editing. VL: Investigation, Methodology, Writing – review & editing, Funding acquisition. MA: Funding acquisition, Investigation, Methodology, Writing – review & editing, Conceptualization, Project administration, Resources, Supervision. DS: Funding acquisition, Project administration, Resources, Supervision, Writing – review & editing, Writing – original draft. MJ: Funding acquisition, Project administration, Resources, Supervision, Writing – original draft, Writing – review & editing, Conceptualization, Data curation, Formal Analysis, Investigation, Methodology, Validation, Visualization.
