# Supplementary material for: Establishment of a stable transfection and gene targeting system in Babesia divergens
Source: Front Cell Infect Microbiol. 2023 Dec 13;13:1278041. doi: 10.3389/fcimb.2023.1278041 (PMC10753763; doi:10.3389/fcimb.2023.1278041)
Supplement: Supplementary file 1 [file DataSheet_1.pdf]

frontiers

Supplementary Material

Establishment of a stable transfection and gene targeting system in *Babesia divergens*

Eliana F.G Cubillos[1], Pavla Snebergerova[1,2], Sarka Borsodi[1], Dominika Reichensdorferova[1], Viktoriya Levytska[2], Masahito Asada[3], Daniel Sojka[2], Marie Jalovecka[1,2*]

*** Correspondence:** jalovecka@prf.jcu.cz, sojka@paru.cas.cz

Supplementary Table 1. List of primers used in this study.

**Plasmid integration test**	
fw 5'UTR	5' – CCATGAAGACGTTATCTGGCTTGATG – 3'
rev 5'UTR	5' – GCCTGACAATCACTATCTTTC – 3'
fw 3'UTR	5' – ATGGTTGGTTCGCTAAACTGCAT – 3'
rev 3'UTR	5' – CAACTTCCTTGAAGTTGTCTCCATCCG – 3'

**qPCR**	
fw *gfp*	5' – ACGTATCCCTCTGGCATAGC – 3'
rev *gfp*	5' – GGTGAAGGAGAAGGTGATGC – 3'
fw *hdhfr*	5' – AATGTGTTCGAACCGGCATC – 3'
rev *hdhfr*	5' – TGAATCACCCAGGCCATCTT – 3'
fw *gapdh*	5' – TACTTACGAGCAGATCGTTGC – 3'
rev *gapdh*	5' – CGGCCTTGACATCGAAAATG – 3'

UTR = untranslated region; *gfp* = green fluorescent protein; *hdhfr* = human dihydrofolate reductase; *gapdh* = glyceraldehyde-3-phosphate dehydrogenase

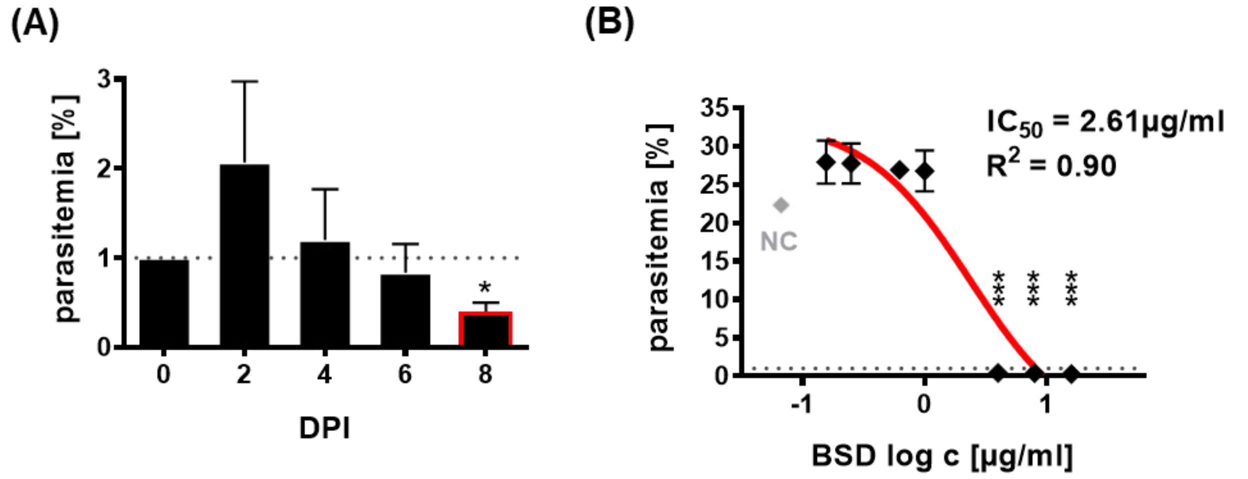

Supplementary Figure 1. Blasticidin S (BSD) as selection drug in *B. divergens in vitro* system. (A) The inhibitory effect of BSD c= 4 µg/ml on *B. divergens in vitro* growth. (B) The IC_{50} value for BSD as determined using non-linear regression with a dose-response curve and regression factor (R^2) based on *B. divergens* parasitemia levels on 8 DPI. Individual concentrations were transformed (log c) prior to analysis. The result represents the mean of three independent replicates, with error bars indicating standard deviations. The grey dotted line represents the initial parasitemia (1%). NC – non-treated culture; IC50 – half-maximal inhibitory concentration; DPI = days post infection; * = $p < 0.05$;*** = $p < 0.001$. One-way ANOVA was performed for statistical analysis.

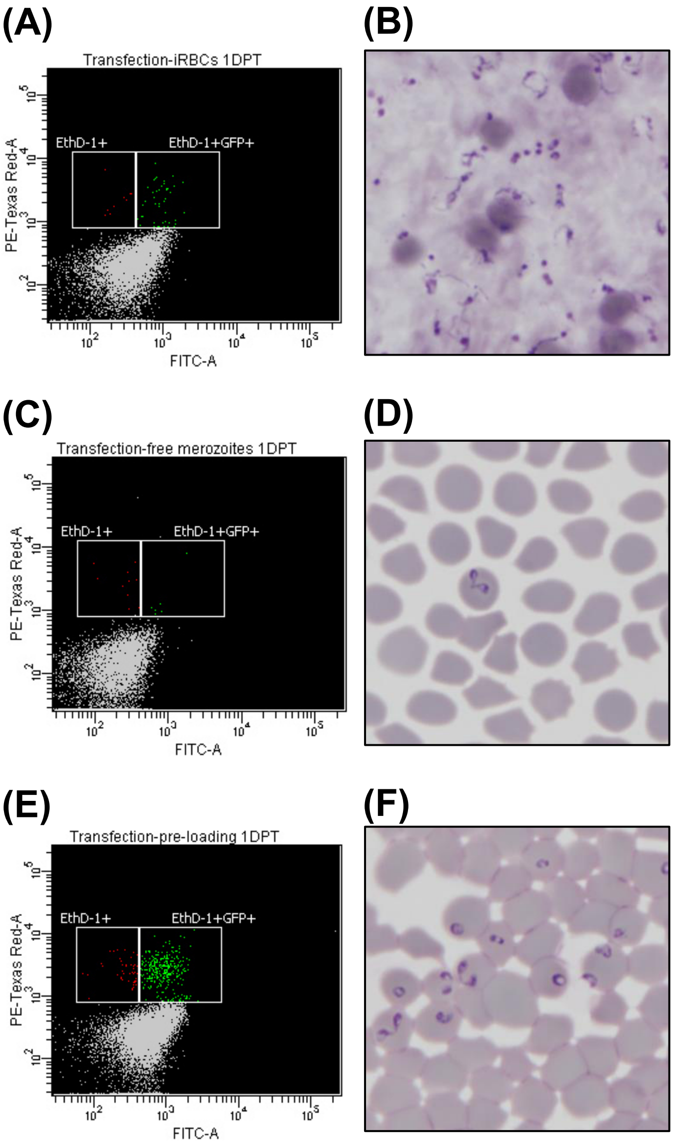

Supplementary Figure 2. Evaluation of different transfection protocols in *B. divergens in vitro* system. (A-B) Direct transfection of iRBCs: (A) The GFP signal detection and (B) appearance of transfected parasite culture on 1 DPT. **(C-D) Electroporation of free merozoites:** (C) The GFP signal detection and (D) appearance of transfected parasite culture on 1 DPT. (**E-F) Plasmid delivery into uRBCs prior to their infection:** (E) The GFP signal detection and (F) appearance of transfected parasite culture on 1 DPT. Culture samples were fixed, stained with EthD-1 to determine total parasitemia, and labeled with an Anti-GFP Polyclonal Antibody conjugated with Alexa Fluor™ 488 to assess GFP signal from parasites using flow cytometry. Thin blood smears were stained with Diff-Quik. DPT = days post transfection; iRBCs = *B. divergens* infected red blood cells; uRBCs = uninfected RBCs; EthD-1 = Ethidium Homodimer 1; GFP = green fluorescent protein.

Suplementary Data

The following section provides detailed maps of the plasmids used in this study. The individual features, such as 5' UTRs, 3' UTRs, gene coding sequences, and restriction sites, are clearly highlighted. Plasmids names: ***act-gfp-ef-tgtp-hdhfr, gfp-ef-tgtp-hdhfr*** and ***6-cys-e-gfp-ef-tgtp-hdhfr.***

Alias: act-gfp-ef-tgtp-hdhfr

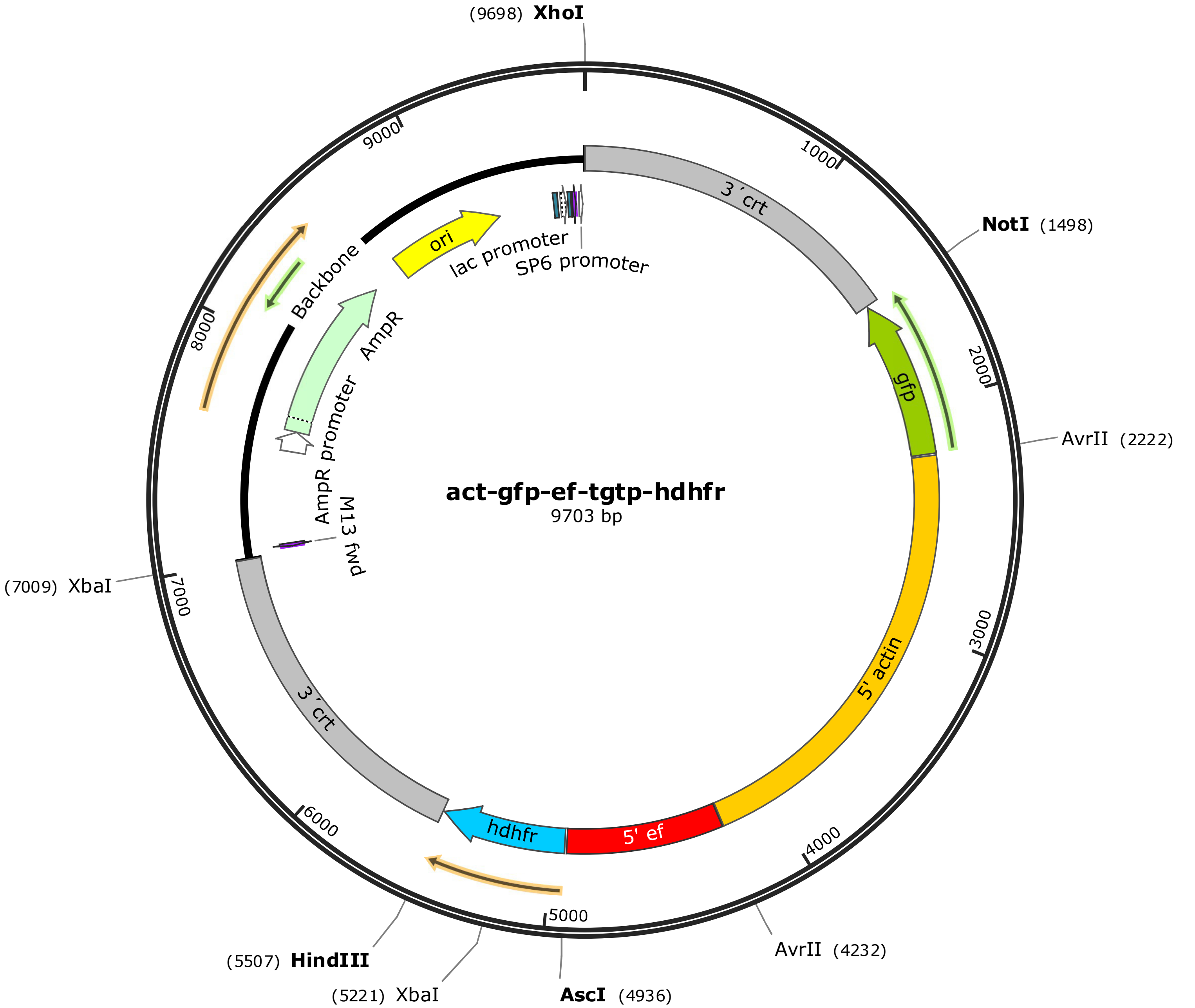

Sequence: 1. act-gfp-ef-tgtp-hdhfr.dna (Circular / 9703 bp)
Enzymes: < Unsaved Enzyme Set > (7 of 678 total) Unique Cutters **Bold**
Features: 16 total

5′ CTAAGGGAGTGTGACTTACCTTCTCAAGGACGTTCTGTGTATAGTAGCCTATAAATATGTCATTAGACACACTAGCATAGCATACCCTGTATGTTTCCAGCTGGGTGAAA 110
3′ GATTCCCTCACACTGAATGGAAGAGTTCCTGCAAGACACATATCATCGGATATTTATACAGTAATCTGTGTGATCGTATCGTATGGGACATACAAAGGTCGACCCACTTT
3´crt

ACGACAGACAAGGATCAACCGCTCCTTATCGCAGCAAGCAACTCCGCAACCAACATCCTGTGGCATATATAATATGTAACAGGGAGTGCTAAGGTACCTTTGAGGACTTC 220
TGCTGTCTGTTCCTAGTTGGCGAGGAATAGCGTCGTTCGTTGAGGCGTTGGTTGTAGGACACCGTATATATTATACATTGTCCCTCACGATTCCATGGAAACTCCTGAAG
3´crt

CAAACGAGCTGCGTAAACGACAAAACATTGGGATTACGGTTCTCAGGACCGCCTGCAATGATGGTCAAACACTATTAACTCTCGTAACCCACCTTCCTGGAAGTCGTAAT 330
GTTTGCTCGACGCATTTGCTGTTTTGTAACCCTAATGCCAAGAGTCCTGGCGGACGTTACTACCAGTTTGTGATAATTGAGAGCATTGGGTGGAAGGACCTTCAGCATTA
3´crt

CACTGTGGCCCTCGTACCAGAACCTGGCAGCGAGCTCCTCCGTGAAGGATGCGACGTCCGATGCAACGTAGTTGGTCCCAATCTCATCACGGTAATACAGCGGAAGGTTG 440
GTGACACCGGGAGCATGGTCTTGGACCGTCGCTCGAGGAGGCACTTCCTACGCTGCAGGCTACGTTGCATCAACCAGGGTTAGAGTAGTGCCATTATGTCGCCTTCCAAC
3´crt

CAACTAACGCGGCGGCTCAGCTACGACAAGTGAAGACGGATTTGGTAACGCTAACCTCGTTCGCTGACCTGCGAGCGTCTGACGCTAGTTTGCTGCTCCTGAACATCGTG 550
GTTGATTGCGCCGCCGAGTCGATGCTGTTCACTTCTGCCTAAACCATTGCGATTGGAGCAAGCGACTGGACGCTCGCAGACTGCGATCAAACGACGAGGACTTGTAGCAC
3´crt

ATCCACGAAACGAAACCGTTGCATCCTACCATTCGAGATGTGGTGACGAATGAAAATCACGGTAGCTATTGAGTGCAGCAAGCATTCTTTCCGTCTAAAACAATATTATT 660
TAGGTGCTTTGCTTTGGCAACGTAGGATGGTAAGCTCTACACCACTGCTTACTTTTAGTGCCATCGATAACTCACGTCGTTCGTAAGAAAGGCAGATTTTGTTATAATAA
3´crt

TCCCCATTAGGGTTTGGCTATCATGAAACCAAACATGTTCACAGAAGGGCTGAGCTCCGTGAATAATACAACACGGGGCTGGATATCAGAATAGCTTATCACACTTGAAA 770
AGGGGTAATCCCAAACCGATAGTACTTTGGTTTGTACAAGTGTCTTCCCGACTCGAGGCACTTATTATGTTGTGCCCCGACCTATAGTCTTATCGAATAGTGTGAACTTT
3´crt

CAACACCTTCATCCTCATATGTGGAACGCGTGAAAGCCATAGTGACACTGTTAGCCAAGGAGCCCCGTGCAAACAACGCGGCTTAAACGCCCAAATAATCTGCAGCCATG 880
GTTGTGGAAGTAGGAGTATACACCTTGCGCACTTTCGGTATCACTGTGACAATCGGTTCCTCGGGGCACGTTTGTTGCGCCGAATTTGCGGGTTTATTAGACGTCGGTAC
3´crt

AACACTTACATCAATGGAGCAAGCAGGAAGATGCCGCGTAAACTGATCATCATCTTCCTCCTCAGATACACAGTTAACCTGTTTATTGACAACCAGCAGAAGCGTTAACC 990
TTGTGAATGTAGTTACCTCGTTCGTCCTTCTACGGCGCATTTGACTAGTAGTAGAAGGAGGAGTCTATGTGTCAATTGGACAAATAACTGTTGGTCGTCTTCGCAATTGG
3´crt

CAAACACACACGCAGCACGTAGCGTAGCCATGACGTGCAACAAAGAAGAGCCGGTTTTCTTACAATTGGAACAACTTAACCGTCAAACTGTTTTCATTTAACATTTCCTA 1100
GTTTGTGTGTGCGTCGTGCATCGCATCGGTACTGCACGTTGTTTCTTCTCGGCCAAAAGAATGTTAACCTTGTTGAATTGGCAGTTTGACAAAAGTAAATTGTAAAGGAT
3´crt

AGGGTCGAGCGCCGACTTACCGGAAGTGGTTCAGGGTAACGCGTGGGAGTTGTACAACGTCCTAAGGAAGGCGATTGTACACTAGGCCACGCACCACATAACACTCATAA 1210
TCCCAGCTCGCGGCTGAATGGCCTTCACCAAGTCCCATTGCGCACCCTCAACATGTTGCAGGATTCCTTCCGCTAACATGTGATCCGGTGCGTGGTGTATTGTGAGTATT
3´crt

GCATATAAGAATCGTCTAGTTTAATAATCGATTTGGTGGCGTTGCACTATGGGACATAGGAAAGCCATGGTGAGCGTATTTGGCCGTAGCACGATGCTACCTAATGCCCG 1320
CGTATATTCTTAGCAGATCAAATTATTAGCTAAACCACCGCAACGTGATACCCTGTATCCTTTCGGTACCACTCGCATAAACCGGCATCGTGCTACGATGGATTACGGGC
3´crt

TGCAAGTGGTCGACGTATACACAACAGGCAAGAACTCACGCAGCGTTCCATCTCCACTAAATATTTAGTGCTACCTCCTATAGTACGTAACAGCAGCTGTACCAATATGT 1430
ACGTTCACCAGCTGCATATGTGTTGTCCGTTCTTGAGTGCGTCGCAAGGTAGAGGTGATTTATAAATCACGATGGAGGATATCATGCATTGTCGTCGACATGGTTATACA
3´crt

NotI
ATCGGTAACCGTGCTAGCCGCTGCAAGAGGCTTTTTGAATTAACTAGCATTAAATCTGATATCGAAGCGGCCGCTTATTTATAAAGCTCATCCATTCCGTGAGTAATACC 1540
TAGCCATTGGCACGATCGGCGACGTTCTCCGAAAAACTTAATTGATCGTAATTTAGACTATAGCTTCGCCGGCGAATAAATATTTCGAGTAGGTAAGGCACTCATTATGG
3´crt gfp
* Lys Tyr Leu Glu Asp Met Gly His Thr Ile Gly

GGCGGCGGTCACGAATTCTAATAAAACCATGTGGTCACGTTTCTCGTTTGGATCCTTACTTAATTTACTTTGTGTAGAAAGATAGTGATTGTCAGGCAACAATACAGGAC 1650
CCGCCGCCAGTGCTTAAGATTATTTTGGTACACCAGTGCAAAGAGCAAACCTAGGAATGAATTAAATGAAACACATCTTTCTATCACTAACAGTCCGTTGTTATGTCCTG
gfp
Ala Ala Thr Val Phe Glu Leu Leu Val Met His Asp Arg Lys Glu Asn Pro Asp Lys Ser Leu Lys Ser Gln Thr Ser Leu Tyr His Asn Asp Pro Leu Leu Val Pro Gly

CATCACCAATAGGTGTATTTTGCTGATAGTGGTCAGCTAATTGCACACTTCCATCCTCAATGTTATGCCTAATCTTAAAGTTTACTTTTATACCATTTTTCTGCTTGTCT 1760
GTAGTGGTTATCCACATAAAACGACTATCACCAGTCGATTAACGTGTGAAGGTAGGAGTTACAATACGGATTAGAATTTCAAATGAAAATATGGTAAAAAGACGAACAGA
gfp
Asp Gly Ile Pro Thr Asn Gln Gln Tyr His Asp Ala Leu Gln Val Ser Gly Asp Glu Ile Asn His Arg Ile Lys Phe Asn Val Lys Ile Gly Asn Lys Gln Lys Asp

GCCATAATATACACATTATGACTGTTGTAATTGTATTCCAACTTATGTCCTAAAATATTACCATCCTCCTTAAAATCAATACCTTTAAGTTCTATCCTGTTAACAAGGGT 1870
CGGTATTATATGTGTAATACTGACAACATTAACATAAGGTTGAATACAGGATTTTATAATGGTAGGAGGAATTTTAGTTATGGAAATTCAAGATAGGACAATTGTTCCCA
gfp
Ala Met Ile Tyr Val Asn His Ser Asn Tyr Asn Tyr Glu Leu Lys His Gly Leu Ile Asn Gly Asp Glu Lys Phe Asp Ile Gly Lys Leu Glu Ile Arg Asn Val Leu Thr

GTCACCCTCAAATTTTACCTCTGCACGAGTTTTGTAGTTACCGTCGTCTTTGAAGAAAATTGTTCTCTCCTGAACGTATCCCTCTGGCATAGCAGACTTAAAGAAGTCGT 1980
CAGTGGGAGTTTAAAATGGAGACGTGCTCAAAACATCAATGGCAGCAGAAACTTCTTTTAACAAGAGAGGACTTGCATAGGGAGACCGTATCGTCTGAATTTCTTCAGCA
gfp
Asp Gly Glu Phe Lys Val Glu Ala Arg Thr Lys Tyr Asn Gly Asp Asp Lys Phe Phe Ile Thr Arg Glu Gln Val Tyr Gly Glu Pro Met Ala Ser Lys Phe Phe Asp His

GTTGCTTCATGTGATCTGGGTAACGTGAGAAGCACTGAACTCCATAAGTCAAGGTGGTAACTAAAGTAGGCCATGGTACTGGAAGTTTTCCTGTAGTGCAAATGAACTTT 2090
CAACGAAGTACACTAGACCCATTGCACTCTTCGTGACTTGAGGTATTCAGTTCCACCATTGATTTCATCCGGTACCATGACCTTCAAAAGGACATCACGTTTACTTGAAA
gfp
Gln Lys Met His Asp Pro Tyr Arg Ser Phe Cys Gln Val Gly Tyr Thr Leu Thr Thr Val Leu Thr Pro Trp Pro Val Pro Leu Lys Gly Thr Thr Cys Ile Phe Lys

AAAGTTAATTTACCGTATGTAGCATCACCTTCTCCTTCACCAGAAACTGAAAATTTGTGACCATTTACATCTCCGTCCAATTCTACAAGAATTGGAACAACTCCAGTAAA 2200
TTTCAATTAAATGGCATACATCGTAGTGGAAGAGGAAGTGGTCTTTGACTTTTAAACACTGGTAAATGTAGAGGCAGGTTAAGATGTTCTTAACCTTGTTGAGGTCATTT
gfp
Leu Thr Leu Lys Gly Tyr Thr Ala Asp Gly Glu Gly Glu Gly Ser Val Ser Phe Lys His Gly Asn Val Asp Gly Asp Leu Glu Val Leu Ile Pro Val Val Gly Thr Phe

AvrII
CAACTCTTCTCCCTTACTCATCCTAGGGGTTTGAAGGGTTATTATCCTAAAAGTGGATGATATGGAGGCAAAAATAAGCGCAAAAACAATTATGATATAACAGCATTTAA 2310
GTTGAGAAGAGGGAATGAGTAGGATCCCCAAACTTCCCAATAATAGGATTTTCACCTACTATACCTCCGTTTTTATTCGCGTTTTTGTTAATACTATATTGTCGTAAATT
gfp 5' actin
Leu Glu Glu Gly Lys Ser Met

CTTATTAGTGGATGTGCACATTGACGCAGTCGACACATAAACCTCTTTCAGTGGGCCGCCGGTCCACCGAGTACCTCAGAATCACCAGTATACACAACAGAAAATGGGTG 2420
GAATAATCACCTACACGTGTAACTGCGTCAGCTGTGTATTTGGAGAAAGTCACCCGGCGGCCAGGTGGCTCATGGAGTCTTAGTGGTCATATGTGTTGTCTTTTACCCAC
5' actin

CATACGTTTTATGCTTTCATGCATTGAGTGCATATACAAAATATGTGAACGGCGCCGGGTGAAATGAATATACCCACGACAACAAGGGGCTGACTCTGGTGTCACAGCAC 2530
GTATGCAAAATACGAAAGTACGTAACTCACGTATATGTTTTATACACTTGCCGCGGCCCACTTTACTTATATGGGTGCTGTTGTTCCCCGACTGAGACCACAGTGTCGTG
5' actin

GTCGATCGGAAAAGGTAAGATGTGAAACAGATATAGTAGATCATCATACGTACCTTGAAATGAGTTTTAAATTGGGCCTTTTGATATGGTGTTGAGTAATCAGAGAGGAA 2640
CAGCTAGCCTTTTCCATTCTACACTTTGTCTATATCATCTAGTAGTATGCATGGAACTTTACTCAAAATTTAACCCGGAAAACTATACCACAACTCATTAGTCTCTCCTT
5' actin

TTACAGTGGTTATTGTGATTGTTCCAATGCACAGGTGGCCACGTTGAAAGGGTGATTCCGTGGGGCTCCCGTCTCTAATCGCGGCAATATTGGTGCCCTTATGCATGGAA 2750
AATGTCACCAATAACACTAACAAGGTTACGTGTCCACCGGTGCAACTTTCCCACTAAGGCACCCCGAGGGCAGAGATTAGCGCCGTTATAACCACGGGAATACGTACCTT
5' actin

TATAATGGCATCTTGTGTATTTACTAATGCCGCATTATGTACCTAATGTGTATCGTAGGCCTAACCTTTTCCACATTAGCCGTTAAAATTTCACGCCACAGTTTGAAAAT 2860
ATATTACCGTAGAACACATAAATGATTACGGCGTAATACATGGATTACACATAGCATCCGGATTGGAAAAGGTGTAATCGGCAATTTTAAAGTGCGGTGTCAAACTTTTA
5' actin

TTAAGATCCAATTTCTCACAAGGCTGACAGCCTTTAGACCACTACAGCTGACCTCTTTTCATATTTTTTTCACACATACATATTAAGTCTCATTATATCGTAATGTGTAG 2970
AATTCTAGGTTAAAGAGTGTTCCGACTGTCGGAAATCTGGTGATGTCGACTGGAGAAAAGTATAAAAAAAGTGTGTATGTATAATTCAGAGTAATATAGCATTACACATC
5' actin

ACCATGTGATTGTTGGTGTATCTTCCATTAAAATTCTATCCATCTGCAAGATCTCGGTTGAATGAAAGAATTCGTGACCTCTTTATTACTATTATCTGCGATGTGTATTA 3080
TGGTACACTAACAACCACATAGAAGGTAATTTTAAGATAGGTAGACGTTCTAGAGCCAACTTACTTTCTTAAGCACTGGAGAAATAATGATAATAGACGCTACACATAAT
5' actin

TTTGCACCTGTATATTATGATCCTTTATAGGTTACTGTCGTTATTGTGTAGTAAGCAATTTAACTATTGTCGAGTAACATAAAATATATTTTGTGGGTGTATCTTCTGTA 3190
AAACGTGGACATATAATACTAGGAAATATCCAATGACAGCAATAACACATCATTCGTTAAATTGATAACAGCTCATTGTATTTTATATAAAACACCCACATAGAAGACAT
5' actin

TACAATTAGTTCAAACATGGCATGTTAACATACCCATTACATGTTTTATTGTTTGGTGGTATGTCGGTTTTCTTGTTTTGGCATTTGGTCATTGATATCACATGTTGTCA 3300
ATGTTAATCAAGTTTGTACCGTACAATTGTATGGGTAATGTACAAAATAACAAACCACCATACAGCCAAAAGAACAAAACCGTAAACCAGTAACTATAGTGTACAACAGT
5' actin

TCTAATAGATTCTTTGTTGAATCTAAGCGTGACATTCATGAATTCATCGATTGCACGTCTTGTGATGTGGCAATGTTGTCCGAAATGAGCTTATCTATAGCCTATGATTT 3410
AGATTATCTAAGAAACAACTTAGATTCGCACTGTAAGTACTTAAGTAGCTAACGTGCAGAACACTACACCGTTACAACAGGCTTTACTCGAATAGATATCGGATACTAAA
5' actin

GTGCCATCTTGAACATTCTTGTCAGCATTGCCTTTGAAGCATCATGCTACATTGTAGATCTGTTGATTGTTGACATGGCCTCATTTTTAATTTGCTCGCATTTTATGCAA 3520
CACGGTAGAACTTGTAAGAACAGTCGTAACGGAAACTTCGTAGTACGATGTAACATCTAGACAACTAACAACTGTACCGGAGTAAAAATTAAACGAGCGTAAAATACGTT
5' actin

CTCAACGGGGCAGCATATATCCCATAGCTGATAAACATATGCATGACTGCATGTTAGCACTGTTAGGTACTATCTGCGGCGGTGCATCATAAAAGCGAATGGCATATGTC 3630
GAGTTGCCCCGTCGTATATAGGGTATCGACTATTTGTATACGTACTGACGTACAATCGTGACAATCCATGATAGACGCCGCCACGTAGTATTTTCGCTTACCGTATACAG
5' actin

CTCAATTCCACGAATAAATGTACAAAGTATCTGCGCATGTGAGAGATTAAATAGGAATTCGTGTAGTAGTCGTGCCATCAATCTATAACTAAGCCCGTTTATAATGCTGG 3740
GAGTTAAGGTGCTTATTTACATGTTTCATAGACGCGTACACTCTCTAATTTATCCTTAAGCACATCATCAGCACGGTAGTTAGATATTGATTCGGGCAAATATTACGACC
5' actin

GGTAATGGTAAAGGAATAAACATTATGGTTGATAAAAAGTTCATGTTGATACAGCCGGTAGCTAAATATAAGTGTAAAGGATCATGGGTATCAGGCATATTACTACAATA 3850
CCATTACCATTTCCTTATTTGTAATACCAACTATTTTTCAAGTACAACTATGTCGGCCATCGATTTATATTCACATTTCCTAGTACCCATAGTCCGTATAATGATGTTAT
5' actin

AACTGTTAAACACTTCCAAAATATGTGATGCATCGATATTGTATTATTATATACAATCGCCAAGTAGGGCCAACACATAGCTCATGCCACAATAAACATTAACGTGGATT 3960
TTGACAATTTGTGAAGGTTTTATACACTACGTAGCTATAACATAATAATATATGTTAGCGGTTCATCCCGGTTGTGTATCGAGTACGGTGTTATTTGTAATTGCACCTAA
5' actin

AATAATACCAGCATTTGAATCTTATGGTGAGATAAGCACACGAATATATATGATACTATAATGCTCTGCTATAACGTGAGAAGAGAAATAAAAGCACATAAAGTGCGACA 4070
TTATTATGGTCGTAAACTTAGAATACCACTCTATTCGTGTGCTTATATATACTATGATATTACGAGACGATATTGCACTCTTCTCTTTATTTTCGTGTATTTCACGCTGT
5' actin

TTAAGTGGTGAATACCAGATATGATAACAGTTGTAATGCTGAAAAATACATGAAGCCATACATGTGCAATGTATATAGCTTATAGAGGAAACATTAACGCTAAAAACATC 4180
AATTCACCACTTATGGTCTATACTATTGTCAACATTACGACTTTTTATGTACTTCGGTATGTACACGTTACATATATCGAATATCTCCTTTGTAATTGCGATTTTTGTAG
5' actin

AvrII
TAGCATGGCTGGAATTACTGCCTTCTATGAATATGCGCATAGAGAACAAGACCTAGGGTTTGGCACTGTTGCTCCGCCCAGGTGAAGTTACGTAGTCTTATAATGCTGAT 4290
ATCGTACCGACCTTAATGACGGAAGATACTTATACGCGTATCTCTTGTTCTGGATCCCAAACCGTGACAACGAGGCGGGTCCACTTCAATGCATCAGAATATTACGACTA
5' actin
5' ef

CAAAGGAACACTTGTTGAAGCTCCTGTGCCCCGTCATATTCTTTTCACGGGTGTTATTTTTAGCGAATTAGTTTATTTACCTGTATGGGTGGTACTGCGCCTTGCGGTTC 4400
GTTTCCTTGTGAACAACTTCGAGGACACGGGGCAGTATAAGAAAAGTGCCCACAATAAAAATCGCTTAATCAAATAAATGGACATACCCACCATGACGCGGAACGCCAAG
5' ef

CCAACAAGCGTCTCTATTATAGTGGCATGACTACCACGATTAGTTTAGATTCTGCGATTATTTCATCGAGACATGTGTATGTCGAGTAGGTGTTTCATATCTTTCGTTTT 4510
GGTTGTTCGCAGAGATAATATCACCGTACTGATGGTGCTAATCAAATCTAAGACGCTAATAAAGTAGCTCTGTACACATACAGCTCATCCACAAAGTATAGAAAGCAAAA
5' ef

ATCGTTGCGTTTATAGTCACGCGTTACTCCTCTCTGTGCGAGTTCGTTCATTGTATGCTATCCGTCACGGCGTATAACTAGAACGTGCACATAGTGTTTATATATATTAT 4620
TAGCAACGCAAATATCAGTGCGCAATGAGGAGAGACACGCTCAAGCAAGTAACATACGATAGGCAGTGCCGCATATTGATCTTGCACGTGTATCACAAATATATATAATA
5' ef

TTATGTACATTCGTATTAGGGCGCAATATTTGTCTCCTGGCGTGGGTGTTTGTTTCTCTTGGCTGGAGGACAGCTCTGTGCCTTGATGTTATACGTCTGCCTTATATATG 4730
AATACATGTAAGCATAATCCCGCGTTATAAACAGAGGACCGCACCCACAAACAAAGAGAACCGACCTCCTGTCGAGACACGGAACTACAATATGCAGACGGAATATATAC
5' ef

CGCCGCGGTTCGATGTTATGTGAGATATAGTTCCATATTAACGATACAATAGCCTATATCGCCATTACATTTTCCTACTCCGGTATAATTATACTGTAATATGCGTTCTT 4840
GCGGCGCCAAGCTACAATACACTCTATATCAAGGTATAATTGCTATGTTATCGGATATAGCGGTAATGTAAAAGGATGAGGCCATATTAATATGACATTATACGCAAGAA
5' ef

AscI
CACGTTTACATCTTGTGTACTGTAATAAACCTCGCATTTGCATGTATACAGCTGATATTATAATCCTTTGCAGATTAATGGTACCTTGTAATCTGGCGCGCCatggttgg 4950
GTGCAAATGTAGAACACATGACATTATTTGGAGCGTAAACGTACATATGTCGACTATAATATTAGGAAACGTCTAATTACCATGGAACATTAGACCGCGCGGtaccaacc
1
Met Val Gly
5' ef
hdhfr
Met Val Gly

ttcgctaaactgcatcgtcgctgtgtcccagaacatgggcatcggcaagaacggggacctgccctggccaccgctcaggaatgaattcagatatttccagagaatgacca 5060
aagcgatttgacgtagcagcgacacagggtcttgtacccgtagccgttcttgcccctggacgggaccggtggcgagtccttacttaagtctataaaggtctcttactggt
5 10 15 20 25 30 35
Ser Leu Asn Cys Ile Val Ala Val Ser Gln Asn Met Gly Ile Gly Lys Asn Gly Asp Leu Pro Trp Pro Pro Leu Arg Asn Glu Phe Arg Tyr Phe Gln Arg Met Thr
hdhfr
Ser Leu Asn Cys Ile Val Ala Val Ser Gln Asn Met Gly Ile Gly Lys Asn Gly Asp Leu Pro Trp Pro Pro Leu Arg Asn Glu Phe Arg Tyr Phe Gln Arg Met Thr

caacctcttcagtagaaggtaaacagaatctggtgattatgggtaagaagacctggttctccattcctgagaagaatcgacctttaaagggtagaattaatttagttctc 5170
gttggagaagtcatcttccatttgtcttagaccactaatacccattcttctggaccaagaggtaaggactcttcttagctggaaatttcccatcttaattaaatcaagag
40 45 50 55 60 65 70 75
Thr Thr Ser Ser Val Glu Gly Lys Gln Asn Leu Val Ile Met Gly Lys Lys Thr Trp Phe Ser Ile Pro Glu Lys Asn Arg Pro Leu Lys Gly Arg Ile Asn Leu Val Leu
hdhfr
Thr Thr Ser Ser Val Glu Gly Lys Gln Asn Leu Val Ile Met Gly Lys Lys Thr Trp Phe Ser Ile Pro Glu Lys Asn Arg Pro Leu Lys Gly Arg Ile Asn Leu Val Leu

XbaI
agcagagaactcaaggaacctccacaaggagctcatttttctttccagaagtctagatgatgccttaaaacttactgaacaaccagaattagcaaataaagtagacatggt 5280
tcgtctcttgagttccttggaggtgttcctcgagtaaaagaaaggtcttcagatctactacggaattttgaatgacttgttggtcttaatcgtttatttcatctgtacca
80 85 90 95 100 105 110
Ser Arg Glu Leu Lys Glu Pro Pro Gln Gly Ala His Phe Leu Ser Arg Ser Leu Asp Asp Ala Leu Lys Leu Thr Glu Gln Pro Glu Leu Ala Asn Lys Val Asp Met Val
hdhfr
Ser Arg Glu Leu Lys Glu Pro Pro Gln Gly Ala His Phe Leu Ser Arg Ser Leu Asp Asp Ala Leu Lys Leu Thr Glu Gln Pro Glu Leu Ala Asn Lys Val Asp Met Val

ctggatagttggtggcagttctgtttataaggaagccatgaatcacccaggccatcttaaactatttgtgacaaggatcatgcaagactttgaaagtgacacgtttttc 5390
gacctatcaaccaccgtcaagacaaatattccttcggtacttagtgggtccggtagaatttgataaacactgttcctagtacgttctgaaactttcactgtgcaaaaag
115 120 125 130 135 140 145
Trp Ile Val Gly Gly Ser Ser Val Tyr Lys Glu Ala Met Asn His Pro Gly His Leu Lys Leu Phe Val Thr Arg Ile Met Gln Asp Phe Glu Ser Asp Thr Phe Phe
hdhfr
Trp Ile Val Gly Gly Ser Ser Val Tyr Lys Glu Ala Met Asn His Pro Gly His Leu Lys Leu Phe Val Thr Arg Ile Met Gln Asp Phe Glu Ser Asp Thr Phe Phe

cagaaattgatttggagaaatataaacttctgccagaatacccaggtgttctctctgatgtccaggaggagaaaggcattaagtacaaatttgaagtatatgagaagaat 5500
gtctttaactaaacctctttatatttgaagacggtcttatgggtccacaagagagactacaggtcctcctctttccgtaattcatgtttaaacttcatatactcttcta
150 155 160 165 170 175 180 185
Pro Glu Ile Asp Leu Glu Lys Tyr Lys Leu Leu Pro Glu Tyr Pro Gly Val Leu Ser Asp Val Gln Glu Glu Lys Gly Ile Lys Tyr Lys Phe Glu Val Tyr Glu Lys Asn
hdhfr
Pro Glu Ile Asp Leu Glu Lys Tyr Lys Leu Leu Pro Glu Tyr Pro Gly Val Leu Ser Asp Val Gln Glu Glu Lys Gly Ile Lys Tyr Lys Phe Glu Val Tyr Glu Lys Asn

HindIII
gattaaAAGCTTTTCGATATCAGATTTAATGCTAGTTAATTCAAAAAGCCTCTTGCAGCGGCTAGCACGGTTACCGATACATATTGGTACAGCTGCTGTTACGTACTATA 5610
ctaattTTCGAAAAGCTATAGTCTAAATTACGATCAATTAAGTTTTTCGGAGAACGTCGCCGATCGTGCCAATGGCTATGTATAACCATGTCGACGACAATGCATGATAT
Asp *
hdhfr
3´crt
Asp *

GGAGGTAGCACTAAATATTTAGTGGAGATGGAACGCTGCGTGAGTTCTTGCCTGTTGTGTATACGTCGACCACTTGCACGGGCATTAGGTAGCATCGTGCTACGGCCAAA 5720
CCTCCATCGTGATTTATAAATCACCTCTACCTTGCGACGCACTCAAGAACGGACAACACATATGCAGCTGGTGAACGTGCCCGTAATCCATCGTAGCACGATGCCGGTTT
3´crt

TACGCTCACCATGGCTTTCCTATGTCCCATAGTGCAACGCCACCAAATCGATTATTAAACTAGACGATTCTTATATGCTTATGAGTGTTATGTGGTGCGTGGCCTAGTGT 5830
ATGCGAGTGGTACCGAAAGGATACAGGGTATCACGTTGCGGTGGTTTAGCTAATAATTTGATCTGCTAAGAATATACGAATACTCACAATACACCACGCACCGGATCACA
3´crt

ACAATCGCCTTCCTTAGGACGTTGTACAACTCCCACGCGTTACCCTGAACCACTTCCGGTAAGTCGGCGCTCGACCCTTAGGAAATGTTAAATGAAAACAGTTTGACGGT 5940
TGTTAGCGGAAGGAATCCTGCAACATGTTGAGGGTGCGCAATGGGACTTGGTGAAGGCCATTCAGCCGCGAGCTGGGAATCCTTTACAATTTACTTTTGTCAAACTGCCA
3´crt

TAAGTTGTTCCAATTGTAAGAAAACCGGCTCTTCTTTGTTGCACGTCATGGCTACGCTACGTGCTGCGTGTGTGTTTGGGTTAACGCTTCTGCTGGTTGTCAATAAACAG 6050
ATTCAACAAGGTTAACATTCTTTTGGCCGAGAAGAAACAACGTGCAGTACCGATGCGATGCACGACGCACACACAAACCCAATTGCGAAGACGACCAACAGTTATTTGTC
3´crt

GTTAACTGTGTATCTGAGGAGGAAGATGATGATCAGTTTACGCGGCATCTTCCTGCTTGCTCCATTGATGTAAGTGTTCATGGCTGCAGATTATTTGGGCGTTTAAGCCG 6160
CAATTGACACATAGACTCCTCCTTCTACTACTAGTCAAATGCGCCGTAGAAGGACGAACGAGGTAACTACATTCACAAGTACCGACGTCTAATAAACCCGCAAATTCGGC
3´crt

CGTTGTTTGCACGGGGCTCCTTGGCTAACAGTGTCACTATGGCTTTCACGCGTTCCACATATGAGGATGAAGGTGTTGTTTCAAGTGTGATAAGCTATTCTGATATCCAG 6270
GCAACAAACGTGCCCCGAGGAACCGATTGTCACAGTGATACCGAAAGTGCGCAAGGTGTATACTCCTACTTCCACAACAAAGTTCACACTATTCGATAAGACTATAGGTC
3´crt

CCCCGTGTTGTATTATTCACGGAGCTCAGCCCTTCTGTGAACATGTTTGGTTTCATGATAGCCAAACCCTAATGGGGAAATAATATTGTTTTAGACGGAAAGAATGCTTG 6380
GGGGCACAACATAATAAGTGCCTCGAGTCGGGAAGACACTTGTACAAACCAAAGTACTATCGGTTTGGGATTACCCCTTTATTATAACAAAATCTGCCTTTCTTACGAAC
3´crt

CTGCACTCAATAGCTACCGTGATTTTCATTCGTCACCACATCTCGAATGGTAGGATGCAACGGTTTCGTTTCGTGGATCACGATGTTCAGGAGCAGCAAACTAGCGTCAG 6490
GACGTGAGTTATCGATGGCACTAAAAGTAAGCAGTGGTGTAGAGCTTACCATCCTACGTTGCCAAAGCAAAGCACCTAGTGCTACAAGTCCTCGTCGTTTGATCGCAGTC
3´crt

ACGCTCGCAGGTCAGCGAACGAGGTTAGCGTTACCAAATCCGTCTTCACTTGTCGTAGCTGAGCCGCCGCGTTAGTTGCAACCTTCCGCTGTATTACCGTGATGAGATTG 6600
TGCGAGCGTCCAGTCGCTTGCTCCAATCGCAATGGTTTAGGCAGAAGTGAACAGCATCGACTCGGCGGCGCAATCAACGTTGGAAGGCGACATAATGGCACTACTCTAAC
3´crt

GGACCAACTACGTTGCATCGGACGTCGCATCCTTCACGGAGGAGCTCGCTGCCAGGTTCTGGTACGAGGGCCACAGTGATTACGACTTCCAGGAAGGTGGGTTACGAGAG 6710
CCTGGTTGATGCAACGTAGCCTGCAGCGTAGGAAGTGCCTCCTCGAGCGACGGTCCAAGACCATGCTCCCGGTGTCACTAATGCTGAAGGTCCTTCCACCCAATGCTCTC
3´crt

TTAATAGTGTTTGACCATCATTGCAGGCGGTCCTGAGAACCGTAATCCCAATGTTTTGTCGTTTACGCAGCTCGTTTGGAAGTCCTCAAAGGTACCTTAGCACTCCCTGT 6820
AATTATCACAAACTGGTAGTAACGTCCGCCAGGACTCTTGGCATTAGGGTTACAAAACAGCAAATGCGTCGAGCAAACCTTCAGGAGTTTCCATGGAATCGTGAGGGACA
3´crt

TACATATTATATATGCCACAGGATGTTGGTTGCGGAGTTGCTTGCTGCGATAAGGAGCGGTTGATCCTTGTCTGTCGTTTTCACCCAGCTGGAAACATACAGGGTATGCT 6930
ATGTATAATATATACGGTGTCCTACAACCAACGCCTCAACGAACGACGCTATTCCTCGCCAACTAGGAACAGACAGCAAAAGTGGGTCGACCTTTGTATGTCCCATACGA
3´crt

XbaI
ATGCTAGTGTGTCTAATGACATATTTTATAGGCTACTATACACAGAACGTCCTTGAGAAGGTAAGTCACACTCCCTTAGTCTAGActatagtgagtcgtattacaattcac 7040
TACGATCACACAGATTACTGTATAAAATATCCGATGATATGTGTCTTGCAGGAACTCTTCCATTCAGTGTGAGGGAATCAGATCTgatatcactcagcataatgttaagtg
3´crt
Backbone
M13 fwd

tggccgtcgttttacaacgtcgtgactgggaaaaccctggcgttacccaacttaatcgccttgcagcacatccccctttcgccagctggcgtaatagcgaagaggcccgc 7150
accggcagcaaaatgttgcagcactgacccttttgggaccgcaatgggttgaattagcggaacgtcgtgtagggggaaagcggtcgaccgcattatcgcttctccgggcg
Backbone
M13 fwd

accgatcgcccttcccaacagttgcgcagcctgaatggcgaatggcgcctgatgcggtattttctccttacgcatctgtgcggtatttcacaccgcatatggtgcactct 7260
tggctagcgggaagggttgtcaacgcgtcggacttaccgcttaccgcggactacgccataaaagaggaatgcgtagacacgccataaagtgtggcgtataccacgtgaga
Backbone

cagtacaatctgctctgatgccgcatagttaagccagccccgacacccgccaacacccgctgacgcgccctgacgggcttgtctgctcccggcatccgcttacagacaag 7370
gtcatgttagacgagactacggcgtatcaattcggtcggggctgtgggcggttgtgggcgactgcgcgggactgcccgaacagacgagggccgtaggcgaatgtctgttc
Backbone

ctgtgaccgtctccgggagctgcatgtgtcagaggttttcaccgtcatcaccgaaacgcgcgagacgaaagggcctcgtgatacgcctatttttataggttaatgtcatg 7480
gacactggcagaggccctcgacgtacacagtctccaaaagtggcagtagtggctttgcgcgctctgctttcccggagcactatgcggataaaaatatccaattacagtac
Backbone

ataataatggtttcttagacgtcaggtggcacttttcggggaaatgtgcgcggaacccctatttgtttatttttctaaatacattcaaatatgtatccgctcatgagaca 7590
tattattaccaaagaatctgcagtccaccgtgaaaagcccctttacacgcgccttggggataaacaaataaaaagatttatgtaagtttatacataggcgagtactctgt
Backbone

AmpR promoter

ataaccctgataaatgcttcaataatattgaaaaaggaagagtatgagtattcaacatttccgtgtcgcccttattcccttttttgcggcattttgccttcctgtttttg 7700
tattgggactatttacgaagttattataactttttccttctcatactcataagttgtaaaggcacagcgggaataagggaaaaaacgccgtaaaacggaaggacaaaaac
Backbone

AmpR promoter

1 Met Ser Ile Gln 5 His Phe Arg Val Ala 10 Leu Ile Pro Phe Phe 15 Ala Ala Phe Cys Leu 20 Pro Val Phe
signal sequence
AmpR

Met Ser Ile Gln His Phe Arg Val Ala Leu Ile Pro Phe Phe Ala Ala Phe Cys Leu Pro Val Phe

ctcacccagaaacgctggtgaaagtaaaagatgctgaagatcagttgggtgcacgagtgggttacatcgaactggatctcaacagcggtaagatccttgagagttttcgc 7810
gagtgggtctttgcgaccactttcattttctacgacttctagtcaacccacgtgctcacccaatgtagcttgacctagagttgtcgccattctaggaactctcaaaagcg
Backbone

Ala His 25 Pro Glu Thr Leu Val 30 Lys Val Lys Asp Ala 35 Glu Asp Gln Leu Gly 40 Ala Arg Val Gly Tyr 45 Ile Glu Leu Asp Leu 50 Asn Ser Gly Lys Ile 55 Leu Glu Ser Phe Arg
AmpR

Ala His Pro Glu Thr Leu Val Lys Val Lys Asp Ala Glu Asp Gln Leu Gly Ala Arg Val Gly Tyr Ile Glu Leu Asp Leu Asn Ser Gly Lys Ile Leu Glu Ser Phe Arg

cccgaagaacgttttccaatgatgagcacttttaaagttctgctatgtggcgcggtattatcccgtattgacgccgggcaagagcaactcggtcgccgcatacactattc 7920
gggcttcttgcaaaaggttactactcgtgaaaatttcaagacgatacaccgcgccataatagggcataactgcggcccgttctcgttgagccagcggcgtatgtgataag
Backbone

60 Pro Glu Glu Arg Phe 65 Pro Met Met Ser Thr 70 Phe Lys Val Leu Leu 75 Cys Gly Ala Val Leu 80 Ser Arg Ile Asp Ala 85 Gly Gln Glu Gln Leu 90 Gly Arg Arg Ile His 95 Tyr Ser
AmpR

Pro Glu Glu Arg Phe Pro Met Met Ser Thr Phe Lys Val Leu Leu Cys Gly Ala Val Leu Ser Arg Ile Asp Ala Gly Gln Glu Gln Leu Gly Arg Arg Ile His Tyr Ser

tcagaatgacttggttgagtactcaccagtcacagaaaagcatcttacggatggcatgacagtaagagaattatgcagtgctgccataaccatgagtgataacactgcgg 8030
agtcttactgaaccaactcatgagtggtcagtgtcttttcgtagaatgcctaccgtactgtcattctcttaatacgtcacgacggtattggtactcactattgtgacgcc
Backbone

Gln Asn Asp 100 Leu Val Glu Tyr Ser 105 Pro Val Thr Glu Lys 110 His Leu Thr Asp Gly 115 Met Thr Val Arg Glu 120 Leu Cys Ser Ala Ala 125 Ile Thr Met Ser Asp 130 Asn Thr Ala
AmpR

Gln Asn Asp Leu Val Glu Tyr Ser Pro Val Thr Glu Lys His Leu Thr Asp Gly Met Thr Val Arg Glu Leu Cys Ser Ala Ala Ile Thr Met Ser Asp Asn Thr Ala

ccaacttacttctgacaacgatcggaggaccgaaggagctaaccgcttttttgcacaacatgggggatcatgtaactcgccttgatcgttgggaaccggagctgaatgaa 8140
ggttgaatgaagactgttgctagcctcctggcttcctcgattggcgaaaaaacgtgttgtaccccctagtacattgagcggaactagcaacccttggcctcgacttactt
Backbone

Ala Asn 135 Leu Leu Leu Thr Thr 140 Ile Gly Gly Pro Lys 145 Glu Leu Thr Ala Phe 150 Leu His Asn Met Gly 155 Asp His Val Thr Arg 160 Leu Asp Arg Trp Glu 165 Pro Glu Leu Asn Glu
AmpR

Ala Asn Leu Leu Leu Thr Thr Ile Gly Gly Pro Lys Glu Leu Thr Ala Phe Leu His Asn Met Gly Asp His Val Thr Arg Leu Asp Arg Trp Glu Pro Glu Leu Asn Glu
* Thr Val Arg Arg Ser Arg Gln Ser Gly Ser Ser Phe Ser

gccataccaaacgacgagcgtgacaccacgatgcctgtagcaatgccaacaacgttgcgcaaactattaactggcgaactacttactctagcttcccggcaacaattaat 8250
cggtatggtttgctgctcgcactgtggtgctacggacatcgttacggttgttgcaacgcgtttgataattgaccgcttgatgaatgagatcgaagggccgttgttaatta
Backbone

170 Ala Ile Pro Asn Asp 175 Glu Arg Asp Thr Thr 180 Met Pro Val Ala Met 185 Pro Thr Thr Leu Arg 190 Lys Leu Leu Thr Gly 195 Glu Leu Leu Thr Leu 200 Ala Ser Arg Gln Gln 205 Leu Ile
AmpR

Ala Ile Pro Asn Asp Glu Arg Asp Thr Thr Met Pro Val Ala Met Pro Thr Thr Leu Arg Lys Leu Leu Thr Gly Glu Leu Leu Thr Leu Ala Ser Arg Gln Gln Leu Ile
Ala Met Gly Phe Ser Ser Arg Ser Val Val Ile Gly Thr Ala Ile Gly Val Val Asn Arg Leu Ser Asn Val Pro Ser Ser Ser Val Arg Ala Glu Arg Cys Cys Asn Ile

agactggatggaggcggataaagttgcaggaccacttctgcgctcggcccttccggctggctggtttattgctgataaatctggagccggtgagcgtgggtctcgcggta 8360
tctgacctacctccgcctatttcaacgtcctggtgaagacgcgagccgggaaggccgaccgaccaaataacgactatttagacctcggccactcgcacccagagcgccat
Backbone

Asp Trp Met 210 Glu Ala Asp Lys Val 215 Ala Gly Pro Leu Leu 220 Arg Ser Ala Leu Pro 225 Ala Gly Trp Phe Ile 230 Ala Asp Lys Ser Gly 235 Ala Gly Glu Arg Gly 240 Ser Arg Gly
AmpR

Asp Trp Met Glu Ala Asp Lys Val Ala Gly Pro Leu Leu Arg Ser Ala Leu Pro Ala Gly Trp Phe Ile Ala Asp Lys Ser Gly Ala Gly Glu Arg Gly Ser Arg Gly
Ser Gln Ile Ser Ala Ser Leu Thr Ala Pro Gly Ser Arg Arg Glu Ala Arg Gly Ala Pro Gln Asn Ile Ala Ser Leu Asp Pro Ala Pro Ser Arg Pro Asp Arg Pro Ile

tcattgcagcactggggccagatggtaagccctcccgtatcgtagttatctacacgacggggagtcaggcaactatggatgaacgaaatagacagatcgctgagataggt 8470
agtaacgtcgtgaccccggtctaccattcgggagggcatagcatcaatagatgtgctgcccctcagtccgttgatacctacttgctttatctgtctagcgactctatcca
Backbone

245 250 255 260 265 270 275
Ile Ile Ala Ala Leu Gly Pro Asp Gly Lys Pro Ser Arg Ile Val Val Ile Tyr Thr Thr Gly Ser Gln Ala Thr Met Asp Glu Arg Asn Arg Gln Ile Ala Glu Ile Gly
AmpR
Ile Ile Ala Ala Leu Gly Pro Asp Gly Lys Pro Ser Arg Ile Val Val Ile Tyr Thr Thr Gly Ser Gln Ala Thr Met Asp Glu Arg Asn Arg Gln Ile Ala Glu Ile Gly
Met

gcctcactgattaagcattggtaactgtcagaccaagtttactcatatatactttagattgatttaaaacttcatttttaatttaaaaggatctaggtgaagatcctttt 8580
cggagtgactaattcgtaaccattgacagtctggttcaaatgagtatatatgaaatctaactaaattttgaagtaaaaattaaattttcctagatccacttctaggaaaa
Backbone

280 285
Ala Ser Leu Ile Lys His Trp *
AmpR
Ala Ser Leu Ile Lys His Trp *

tgataatctcatgaccaaaatcccttaacgtgagttttcgttccactgagcgtcagaccccgtagaaaagatcaaaggatcttcttgagatcctttttttctgcgcgtaa 8690
actattagagtactggttttagggaattgcactcaaaagcaaggtgactcgcagtctggggcatcttttctagtttcctagaagaactctaggaaaaaaagacgcgcatt
Backbone

ori

tctgctgcttgcaaacaaaaaaaccaccgctaccagcggtggtttgtttgccggatcaagagctaccaactctttttccgaaggtaactggcttcagcagagcgcagata 8800
agacgacgaacgtttgtttttttggtggcgatggtcgccaccaaacaaacggcctagttctcgatggttgagaaaaaggcttccattgaccgaagtcgtctcgcgtctat
Backbone

ori

ccaaatactgtccttctagtgtagccgtagttaggccaccacttcaagaactctgtagcaccgcctacatacctcgctctgctaatcctgttaccagtggctgctgccag 8910
ggtttatgacaggaagatcacatcggcatcaatccggtggtgaagttcttgagacatcgtggcggatgtatggagcgagacgattaggacaatggtcaccgacgacggtc
Backbone

ori

tggcgataagtcgtgtcttaccgggttggactcaagacgatagttaccggataaggcgcagcggtcgggctgaacggggggttcgtgcacacagcccagcttggagcgaa 9020
accgctattcagcacagaatggcccaacctgagttctgctatcaatggcctattccgcgtcgccagcccgacttgccccccaagcacgtgtgtcgggtcgaacctcgctt
Backbone

ori

cgacctacaccgaactgagatacctacagcgtgagctatgagaaagcgccacgcttcccgaagggagaaaggcggacaggtatccggtaagcggcagggtcggaacagga 9130
gctggatgtggcttgactctatggatgtcgcactcgatactctttcgcggtgcgaagggcttccctctttccgcctgtccataggccattcgccgtcccagccttgtcct
Backbone

ori

gagcgcacgagggagcttccagggggaaacgcctggtatctttatagtcctgtcgggtttcgccacctctgacttgagcgtcgatttttgtgatgctcgtcaggggggcg 9240
ctcgcgtgctccctcgaaggtccccctttgcggaccatagaaatatcaggacagcccaaagcggtggagactgaactcgcagctaaaaacactacgagcagtccccccgc
Backbone

ori

gagcctatcgaaaaacgccagcaacgcggcctttttacggttcctggccttttgctggccttttgctcacatgttctttcctgcgttatcccctgattctgtggataacc 9350
ctcggatagctttttgcggtcgttgcgccggaaaaatgccaaggaccggaaaacgaccggaaaacgagtgtacaagaaaggacgcaatagggggactaagacacctattgg
Backbone

ori

gtattaccgcctttgagtgagctgataccgctcgccgcagccgaacgaccgagcgcagcgagtcagtgagcgaggaagcggaagagcgcccaatacgcaaaccgcctctc 9460
cataatggcggaaactcactcgactatggcgagcggcgtcggcttgctggctcgcgtcgctcagtcactcgctccttcgccttctcgcgggttatgcgtttggcggagag
Backbone

cccgcgcgttggccgattcattaatgcagctggcacgacaggtttcccgactggaaagcgggcagtgagcgcaacgcaattaatgtgagttagctcactcattaggcacc 9570
gggcgcgcaaccggctaagtaattacgtcgaccgtgctgtccaaagggctgacctttcgcccgtcactcgcgttgcgttaattacactcaatcgagtgagtaatccgtgg
Backbone

CAP binding site

ccaggctttacactttatgcttccggctcgtatgttgtgtggaattgtgagcggataacaatttcacacaggaaacagctatgaccatgattacgccaagctatttaggt 9680
ggtccgaaatgtgaaatacgaaggccgagcatacaacacaccttaacactcgcctattgttaaagtgtgtcctttgtcgatactggtactaatgcggttcgataaatcca
Backbone

-35 -10
lac promoter
lac operator
M13 rev
SP6 promoter

XhoI
gacactatagaatactcCTCGAG 3′
••• 9703
ctgtgatatcttatgagGAGCTC 5′
Backbone

SP6 promoter

Alias: gfp-ef-tgtp-hdhfr

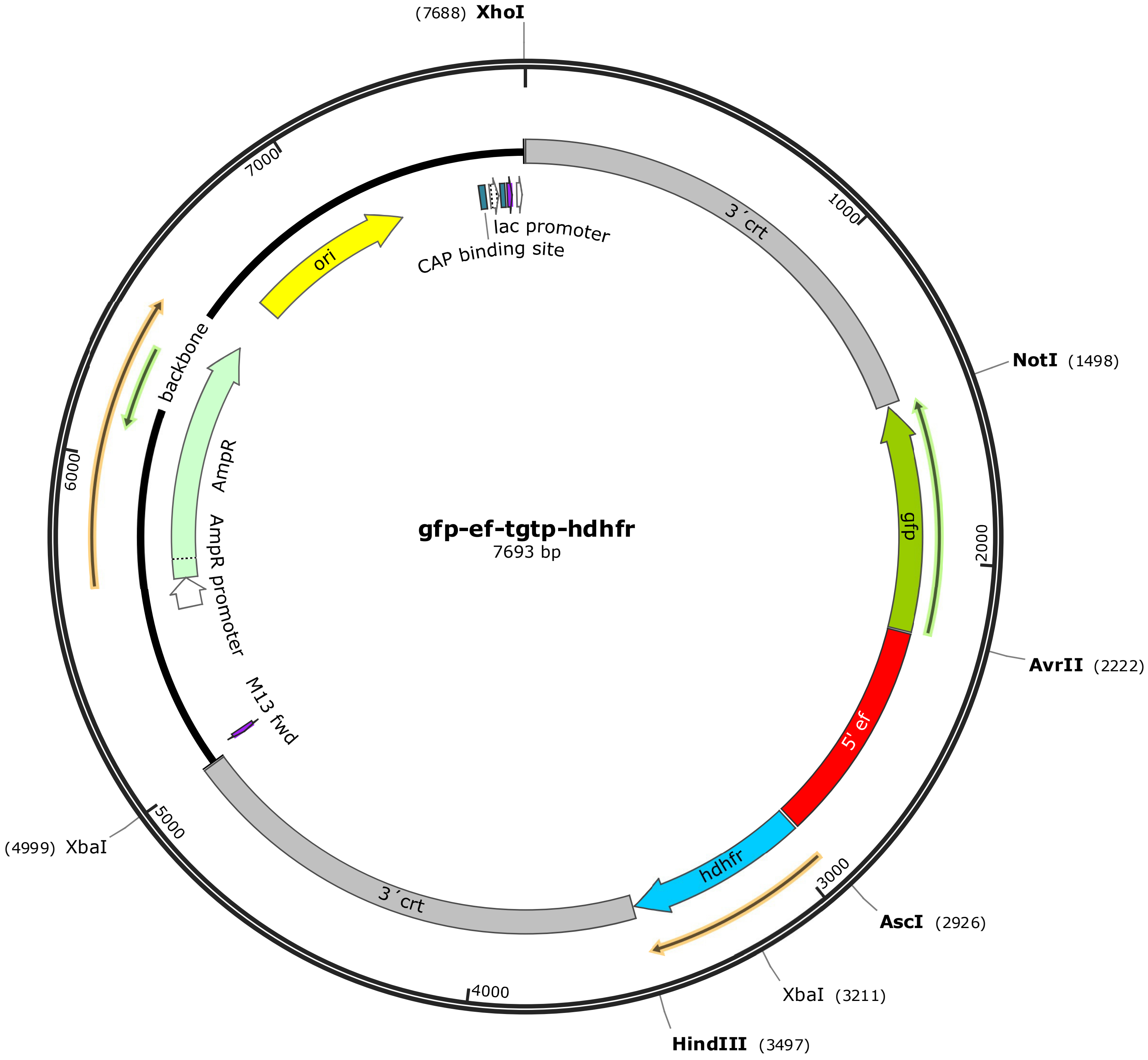

5′ CTAAGGGAGTGTGACTTACCTTCTCAAGGACGTTCTGTGTATAGTAGCCTATAAATATGTCATTAGACACACTAGCATAGCATACCCTGTATGTTTCCAGCTGGGTGAAAACGACAGACAAGGATCAACCGCTCCTTATCGCAGCAAGCAACTCCGCAACCAACATCCTG 170
3′ GATTCCCTCACACTGAATGGAAGAGTTCCTGCAAGACACATATCATCGGATATTTATACAGTAATCTGTGTGATCGTATCGTATGGGACATACAAAGGTCGACCCACTTTTGCTGTCTGTTCCTAGTTGGCGAGGAATAGCGTCGTTCGTTGAGGCGTTGGTTGTAGGAC
3´crt

TGGCATATATAATATGTAACAGGGAGTGCTAAGGTACCTTTGAGGACTTCCAAACGAGCTGCGTAAACGACAAAACATTGGGATTACGGTTCTCAGGACCGCCTGCAATGATGGTCAAACACTATTAACTCTCGTAACCCACCTTCCTGGAAGTCGTAATCACTGTGGCC 340
ACCGTATATATTATACATTGTCCCTCACGATTCCATGGAAACTCCTGAAGGTTTGCTCGACGCATTTGCTGTTTTGTAACCCTAATGCCAAGAGTCCTGGCGGACGTTACTACCAGTTTGTGATAATTGAGAGCATTGGGTGGAAGGACCTTCAGCATTAGTGACACCGG
3´crt

CTCGTACCAGAACCTGGCAGCGAGCTCCTCCGTGAAGGATGCGACGTCCGATGCAACGTAGTTGGTCCCAATCTCATCACGGTAATACAGCGGAAGGTTGCAACTAACGCGGCGGCTCAGCTACGACAAGTGAAGACGGATTTGGTAACGCTAACCTCGTTCGCTGACCT 510
GAGCATGGTCTTGGACCGTCGCTCGAGGAGGCACTTCCTACGCTGCAGGCTACGTTGCATCAACCAGGGTTAGAGTAGTGCCATTATGTCGCCTTCCAACGTTGATTGCGCCGCCGAGTCGATGCTGTTCACTTCTGCCTAAACCATTGCGATTGGAGCAAGCGACTGGA
3´crt

GCGAGCGTCTGACGCTAGTTTGCTGCTCCTGAACATCGTGATCCACGAAACGAAACCGTTGCATCCTACCATTCGAGATGTGGTGACGAATGAAAATCACGGTAGCTATTGAGTGCAGCAAGCATTCTTTCCGTCTAAAACAATATTATTTCCCCATTAGGGTTTGGCTA 680
CGCTCGCAGACTGCGATCAAACGACGAGGACTTGTAGCACTAGGTGCTTTGCTTTGGCAACGTAGGATGGTAAGCTCTACACCACTGCTTACTTTTAGTGCCATCGATAACTCACGTCGTTCGTAAGAAAGGCAGATTTTGTTATAATAAAGGGGTAATCCCAAACCGAT
3´crt

TCATGAAACCAAACATGTTCACAGAAGGGCTGAGCTCCGTGAATAATACAACACGGGGCTGGATATCAGAATAGCTTATCACACTTGAAACAACACCTTCATCCTCATATGTGGAACGCGTGAAAGCCATAGTGACACTGTTAGCCAAGGAGCCCCGTGCAAACAACGCG 850
AGTACTTTGGTTTGTACAAGTGTCTTCCCGACTCGAGGCACTTATTATGTTGTGCCCCGACCTATAGTCTTATCGAATAGTGTGAACTTTGTTGTGGAAGTAGGAGTATACACCTTGCGCACTTTCGGTATCACTGTGACAATCGGTTCCTCGGGGCACGTTTGTTGCGC
3´crt

GCTTAAACGCCCAAATAATCTGCAGCCATGAACACTTACATCAATGGAGCAAGCAGGAAGATGCCGCGTAAACTGATCATCATCTTCCTCCTCAGATACACAGTTAACCTGTTTATTGACAACCAGCAGAAGCGTTAACCCAAACACACACGCGCAGCACGTAGCGTAGCCA 1020
CGAATTTGCGGGTTTATTAGACGTCGGTACTTGTGAATGTAGTTACCTCGTTCGTCCTTCTACGGCGCATTTGACTAGTAGTAGAAGGAGGAGTCTATGTGTCAATTGGACAAATAACTGTTGGTCGTCTTCGCAATTGGGTTTGTGTGTGCGCGTCGTGCATCGCATCGGT
3´crt

TGACGTGCAACAAAGAAGAGCCGGTTTTCTTACAATTGGAACAACTTAACCGTCAAACTGTTTTCATTTAACATTTCCTAAGGGTCGAGCGCCGACTTACCGGAAGTGGTTCAGGGTAACGCGTGGGAGTTGTACAACGTCCTAAGGAAGGCGATTGTACACTAGGCCAC 1190
ACTGCACGTTGTTTCTTCTCGGCCAAAAGAATGTTAACCTTGTTGAATTGGCAGTTTGACAAAAGTAAATTGTAAAGGATTCCCAGCTCGCGGCTGAATGGCCTTCACCAAGTCCCATTGCGCACCCTCAACATGTTGCAGGATTCCTTCCGCTAACATGTGATCCGGTG
3´crt

GCACCACATAACACTCATAAGCATATAAGAATCGTCTAGTTTAATAATCGATTTGGTGGCGTTGCACTATGGGACATAGGAAAGCCATGGTGAGCGTATTTGGCCGTAGCACGATGCTACCTAATGCCCGTGCAAGTGGTCGACGTATACACAACAGGCAAGAACTCACG 1360
CGTGGTGTATTGTGAGTATTCGTATATTCTTAGCAGATCAAATTATTAGCTAAACCACCGCAACGTGATACCCTGTATCCTTTCGGTACCACTCGCATAAACCGGCATCGTGCTACGATGGATTACGGGCACGTTCACCAGCTGCATATGTGTTGTCCGTTCTTGAGTGC
3´crt

NotI
CAGCGTTCCATCTCCACTAAATATTTAGTGCTACCTCCTATAGTACGTAACAGCAGCTGTACCAATATGTATCGGTAACCGTGCTAGCCGCTGCAAGAGGCTTTTTGAATTAACTAGCATTAAATCTGATATCGAAGCGGCCGCTTATTTATAAAGCTCATCCATTCCGT 1530
GTCGCAAGGTAGAGGTGATTTATAAATCACGATGGAGGATATCATGCATTGTCGTCGACATGGTTATACATAGCCATTGGCACGATCGGCGACGTTCTCCGAAAAACTTAATTGATCGTAATTTAGACTATAGCTTCGCCGGCGAATAAATATTTCGAGTAGGTAAGGCA
3´crt gfp
Lys Tyr Leu Glu Asp Met Gly His

GAGTAATACCGGCGGCGGTCACGAATTCTAATAAAACCATGTGGTCACGTTTCTCGTTTGGATCCTTACTTAATTTACTTTGTGTAGAAAGATAGTGATTGTCAGGCAACAATACAGGACCATCACCAATAGGTGTATTTTGCTGATAGTGGTCAGCTAATTGCACACTT 1700
CTCATTATGGCCGCCGCCAGTGCTTAAGATTATTTTGGTACACCAGTGCAAAGAGCAAACCTAGGAATGAATTAAATGAAACACATCTTTCTATCACTAACAGTCCGTTGTTATGTCCTGGTAGTGGTTATCCACATAAAACGACTATCACCAGTCGATTAACGTGTGAA
gfp
Thr Ile Gly Ala Ala Thr Val Phe Glu Leu Leu Val Met His Asp Arg Lys Glu Asn Pro Asp Lys Ser Leu Lys Ser Gln Thr Ser Leu Tyr His Asn Asp Pro Leu Leu Val Pro Gly Asp Gly Ile Pro Thr Asn Gln Gln Tyr His Asp Ala Leu Gln Val Ser

CCATCCTCAATGTTATGCCTAATCTTAAAGTTTACTTTTATACCATTTTTCTGCTTGTCTGCCATAATATACACATTATGACTGTTGTAATTGTATTCCAACTTATGTCCTAAAATATTACCATCCTCCTTAAAATCAATACCTTTAAGTTCTATCCTGTTAACAAGGGT 1870
GGTAGGAGTTACAATACGGATTAGAATTTCAAATGAAAATATGGTAAAAAGACGAACAGACGGTATTATATGTGTAATACTGACAACATTAACATAAGGTTGAATACAGGATTTTATAATGGTAGGAGGAATTTTAGTTATGGAAATTCAAGATAGGACAATTGTTCCCA
gfp
Gly Asp Glu Ile Asn His Arg Ile Lys Phe Asn Val Lys Ile Gly Asn Lys Gln Lys Asp Ala Met Ile Tyr Val Asn His Ser Asn Tyr Asn Tyr Glu Leu Lys His Gly Leu Ile Asn Gly Asp Glu Lys Phe Asp Ile Gly Lys Leu Glu Ile Arg Asn Val Leu Thr

GTCACCCTCAAATTTTACCTCTGCACGAGTTTTGTAGTTACCGTCGTCTTTGAAGAAAATTGTTCTCTCCTGAACGTATCCCTCTGGCATAGCAGACTTAAAGAAGTCGTGTTGCTTCATGTGATCTGGGTAACGTGAGAAGCACTGAACTCCATAAGTCAAGGTGGTAA 2040
CAGTGGGAGTTTAAAATGGAGACGTGCTCAAAACATCAATGGCAGCAGAAACTTCTTTTAACAAGAGAGGACTTGCATAGGGAGACCGTATCGTCTGAATTTCTTCAGCACAACGAAGTACACTAGACCCATTGCACTCTTCGTGACTTGAGGTATTCAGTTCCACCATT
gfp
Asp Gly Glu Phe Lys Val Glu Ala Arg Thr Lys Tyr Asn Gly Asp Asp Lys Phe Phe Ile Thr Arg Glu Gln Val Tyr Gly Glu Pro Met Ala Ser Lys Phe Phe Asp His Gln Lys Met His Asp Pro Tyr Arg Ser Phe Cys Gln Val Gly Tyr Thr Leu Thr Thr Val

CTAAAGTAGGCCATGGTACTGGAAGTTTTCCTGTAGTGCAAATGAACTTTAAAGTTAATTTACCGTATGTAGCATCACCTTCTCCTTCACCAGAAACTGAAAATTTGTGACCATTTACATCTCCGTCCAATTCTACAAGAATTGGAACAACTCCAGTAAACAACTCTTCT 2210
GATTTCATCCGGTACCATGACCTTCAAAAGGACATCACGTTTACTTGAAATTTCAATTAAATGGCATACATCGTAGTGGAAGAGGAAGTGGTCTTTGACTTTTAAACACTGGTAAATGTAGAGGCAGGTTAAGATGTTCTTAACCTTGTTGAGGTCATTTGTTGAGAAGA
gfp
Leu Thr Pro Trp Pro Val Pro Leu Lys Gly Thr Thr Cys Ile Phe Lys Leu Thr Leu Lys Gly Tyr Thr Ala Asp Gly Glu Gly Glu Gly Ser Val Ser Phe Lys His Gly Asn Val Asp Gly Asp Leu Glu Val Leu Ile Pro Val Val Gly Thr Phe Leu Glu Glu

AvrII
CCCTTACTCATCCTAGGGTTTGGCACTGTTGCTCCGCCCAGGTGAAGTTACGTAGTCTTATAATGCTGATCAAAGGAACACTTGTTGAAGCTCCTGTGCCCCGTCATATTCTTTTCACGGGTGTTATTTTTAGCGAATTAGTTTATTTACCTGTATGGGTGGTACTGCGC 2380
GGGAATGAGTAGGATCCCAAACCGTGACAACGAGGCGGGTCCACTTCAATGCATCAGAATATTACGACTAGTTTCCTTGTGAACAACTTCGAGGACACGGGGCAGTATAAGAAAAGTGCCCACAATAAAAATCGCTTAATCAAATAAATGGACATACCCACCATGACGCG
gfp 5' ef
Gly Lys Ser Met

CTTGCGGTTCCCAACAAGCGTCTCTATTATAGTGGCATGACTACCACGATTAGTTTAGATTCTGCGATTATTTCATCGAGACATGTGTATGTCGAGTAGGTGTTTCATATCTTTCGTTTTATCGTTGCGTTTATAGTCACGCGTTACTCCTCTCTGTGCGAGTTCGTTCA 2550
GAACGCCAAGGGTTGTTCGCAGAGATAATATCACCGTACTGATGGTGCTAATCAAATCTAAGACGCTAATAAAGTAGCTCTGTACACATACAGCTCATCCACAAAGTATAGAAAGCAAAATAGCAACGCAAATATCAGTGCGCAATGAGGAGAGACACGCTCAAGCAAGT
5' ef

TTGTATGCTATCCGTCACGGCGTATAACTAGAACGTGCACATAGTGTTTATATATATTATTTATGTACATTCGTATTAGGGCGCAATATTTGTCTCCTGGCGTGGGTGTTTGTTTCTCTTGGCTGGAGGACAGCTCTGTGCCTTGATGTTATACGTCTGCCTTATATATG 2720
AACATACGATAGGCAGTGCCGCATATTGATCTTGCACGTGTATCACAAATATATATAATAAATACATGTAAGCATAATCCCGCGTTATAAACAGAGGACCGCACCCACAAACAAAGAGAACCGACCTCCTGTCGAGACACGGAACTACAATATGCAGACGGAATATATAC
5' ef

CGCCGCGGTTCGATGTTATGTGAGATATAGTTCCATATTAACGATACAATAGCCTATATCGCCATTACATTTTCCTACTCCGGTATAATTATACTGTAATATGCGTTCTTCACGTTTACATCTTGTGTACTGTAATAAACCTCGCATTTGCATGTATACAGCTGATATTA 2890
GCGGCGCCAAGCTACAATACACTCTATATCAAGGTATAATTGCTATGTTATCGGATATAGCGGTAATGTAAAAGGATGAGGCCATATTAATATGACATTATACGCAAGAAGTGCAAATGTAGAACACATGACATTATTTGGAGCGTAAACGTACATATGTCGACTATAAT
5' ef

AscI
TAATCCTTTGCAGATTAATGGTACCTTGTAATCTGGCGCGCCatggttggttcgctaaactgcatcgtcgctgtgtcccagaacatgggcatcggcaagaacggggacctgccctggccaccgctcaggaatgaattcagatatttccagagaatgaccacaacctcttc 3060
ATTAGGAAACGTCTAATTACCATGGAACATTAGaCCGCGCGGtaccaaccaagcgatttgacgtagcagcgacacagggtcttgtacccgtagccgttcttgcccctggacgggaccggtggcgagtccttacttaagtctataaaggtctcttactggtgttggagaag
1 5 10 15 20 25 30 35 40
Met Val Gly Ser Leu Asn Cys Ile Val Ala Val Ser Gln Asn Met Gly Ile Gly Lys Asn Gly Asp Leu Pro Trp Pro Pro Leu Arg Asn Glu Phe Arg Tyr Phe Gln Arg Met Thr Thr Thr Ser Ser
5' ef | hdhfr
Met Val Gly Ser Leu Asn Cys Ile Val Ala Val Ser Gln Asn Met Gly Ile Gly Lys Asn Gly Asp Leu Pro Trp Pro Pro Leu Arg Asn Glu Phe Arg Tyr Phe Gln Arg Met Thr Thr Thr Ser Ser

XbaI
agtagaaggtaaacagaatctggtgattatgggtaagaagacctggttctccattcctgagaagaatcgacctttaaagggtagaattaatttagttctcagcagagaactcaaggaacctccacaaggagctcattttctttccagaagtctagatgatgccttaaaac 3230
tcatcttccatttgtcttagaccactaatacccattcttctggaccaagaggtaaggactcttcttagctggaaatttcccatcttaattaaatcaagagtcgtctcttgagttccttggaggtgttcctcgagtaaaagaaaggtcttcagatctactacggaattttg
45 50 55 60 65 70 75 80 85 90 95
Val Glu Gly Lys Gln Asn Leu Val Ile Met Gly Lys Lys Thr Trp Phe Ser Ile Pro Glu Lys Asn Arg Pro Leu Lys Gly Arg Ile Asn Leu Val Leu Ser Arg Glu Leu Lys Glu Pro Pro Gln Gly Ala His Phe Leu Ser Arg Ser Leu Asp Asp Ala Leu Lys
hdhfr
Val Glu Gly Lys Gln Asn Leu Val Ile Met Gly Lys Lys Thr Trp Phe Ser Ile Pro Glu Lys Asn Arg Pro Leu Lys Gly Arg Ile Asn Leu Val Leu Ser Arg Glu Leu Lys Glu Pro Pro Gln Gly Ala His Phe Leu Ser Arg Ser Leu Asp Asp Ala Leu Lys

ttactgaacaaccagaattagcaaataaagtagacatggtctggatagttggtggcagttctgtttataaggaagccatgaatcacccaggccatcttaaactatttgtgacaaggatcatgcaagactttgaaagtgacacgtttttttccagaaattgatttggagaaa 3400
aatgacttgttggtcttaatcgtttatttcatctgtaccagacctatcaaccaccgtcaagacaaatattccttcggtacttagtgggtccggtagaatttgataaacactgttcctagtacgttctgaaactttcactgtgcaaaaaaaggtctttaactaaacctcttt
100 105 110 115 120 125 130 135 140 145 150 155
Leu Thr Glu Gln Pro Glu Leu Ala Asn Lys Val Asp Met Val Trp Ile Val Gly Gly Ser Ser Val Tyr Lys Glu Ala Met Asn His Pro Gly His Leu Lys Leu Phe Val Thr Arg Ile Met Gln Asp Phe Glu Ser Asp Thr Phe Phe Pro Glu Ile Asp Leu Glu Lys
hdhfr
Leu Thr Glu Gln Pro Glu Leu Ala Asn Lys Val Asp Met Val Trp Ile Val Gly Gly Ser Ser Val Tyr Lys Glu Ala Met Asn His Pro Gly His Leu Lys Leu Phe Val Thr Arg Ile Met Gln Asp Phe Glu Ser Asp Thr Phe Phe Pro Glu Ile Asp Leu Glu Lys

Hind III
tataaacttctgccagaatacccaggtgttctctctgatgtccaggaggagaaaggcattaagtacaaatttgaagtatatgagaagaatgattaaAAGCTTTTCGATATCAGATTTAATGCTAGTTAATTCAAAAAGCCTCTTGCAGCGGCTAGCACGGTTACCGATAC 3570
atatttgaagacggtcttatgggtccacaagagagactacaggtcctcctctttccgtaattcatgtttaaacttcatatactcttcttactaattTTCGAAAAGCTATAGTCTAAATTACGATCAATTAAGTTTTTCGGAGAACGTCGCCGATCGTGCCAATGGCTATG
160 165 170 175 180 185
Tyr Lys Leu Leu Pro Glu Tyr Pro Gly Val Leu Ser Asp Val Gln Glu Glu Lys Gly Ile Lys Tyr Lys Phe Glu Val Tyr Glu Lys Asn Asp *
hdhfr | 3´crt
Tyr Lys Leu Leu Pro Glu Tyr Pro Gly Val Leu Ser Asp Val Gln Glu Glu Lys Gly Ile Lys Tyr Lys Phe Glu Val Tyr Glu Lys Asn Asp *

ATATTGGTACAGCTGCTGTTACGTACTATAGGAGGTAGCACTAAATATTTAGTGGAGATGGAACGCTGCGTGAGTTCTTGCCTGTTGTGTATACGTCGACCACTTGCACGGGCATTAGGTAGCATCGTGCTACGGCCAAATACGCTCACCATGGCTTTCCTATGTCCCAT 3740
TATAACCATGTCGACGACAATGCATGATATCCTCCATCGTGATTTATAAATCACCTCTACCTTGCGACGCACTCAAGAACGGACAACACATATGCAGCTGGTGAACGTGCCCGTAATCCATCGTAGCACGATGCCGGTTTATGCGAGTGGTACCGAAAGGATACAGGGTA
3´crt

AGTGCAACGCCACCAAATCGATTATTAAACTAGACGATTCTTATATGCTTATGAGTGTTATGTGGTGCGTGGCCTAGTGTACAATCGCCTTCCTTAGGACGTTGTACAACTCCCACGCGTTACCCTGAACCACTTCCGGTAAGTCGGCGCTCGACCCTTAGGAAATGTTA 3910
TCACGTTGCGGTGGTTTAGCTAATAATTTGATCTGCTAAGAATATACGAATACTCACAATACACCACGCACCGGATCACATGTTAGCGGAAGGAATCCTGCAACATGTTGAGGGTGCGCAATGGGACTTGGTGAAGGCCATTCAGCCGCGAGCTGGGAATCCTTTACAAT
3´crt

AATGAAAACAGTTTGACGGTTAAGTTGTTCCAATTGTAAGAAAACCGGCTCTTCTTTGTTGCACGTCATGGCTACGCTACGTGCTGCGTGTGTGTTTGGGTTAACGCTTCTGCTGGTTGTCAATAAACAGGTTAACTGTGTATCTGAGGAGGAAGATGATGATCAGTTTA 4080
TTACTTTTGTCAAACTGCCAATTCAACAAGGTTAACATTCTTTTGGCCGAGAAGAAACAACGTGCAGTACCGATGCGATGCACGACGCACACACAAACCCAATTGCGAAGACGACCAACAGTTATTTGTCCAATTGACACATAGACTCCTCCTTCTACTACTAGTCAAAT
3´crt

CGCGGCATCTTCCTGCTTGCTCCATTGATGTAAGTGTTCATGGCTGCAGATTATTTGGGCGTTTAAGCCGCGTTGTTTGCACGGGGCTCCTTGGCTAACAGTGTCACTATGGCTTTCACGCGTTCCACATATGAGGATGAAGGTGTTGTTTCAAGTGTGATAAGCTATTC 4250
GCGCCGTAGAAGGACGAACGAGGTAACTACATTCACAAGTACCGACGTCTAATAAACCCGCAAATTCGGCGCAACAAACGTGCCCCGAGGAACCGATTGTCACAGTGATACCGAAAGTGCGCAAGGTGTATACTCCTACTTCCACAACAAAGTTCACACTATTCGATAAG
3´crt

TGATATCCAGCCCCGTGTTGTATTATTCACGGAGCTCAGCCCTTCTGTGAACATGTTTGGTTTCATGATAGCCAAACCCTAATGGGGAAATAATATTGTTTTAGACGGAAAGAATGCTTGCTGCACTCAATAGCTACCGTGATTTTCATTCGTCACCACATCTCGAATGG 4420
ACTATAGGTCGGGGCACAACATAATAAGTGCCTCGAGTCGGGAAGACACTTGTACAAACCAAAGTACTATCGGTTTGGGATTACCCCTTTATTATAACAAAATCTGCCTTTCTTACGAACGACGTGAGTTATCGATGGCACTAAAAGTAAGCAGTGGTGTAGAGCTTACC
3´crt

TAGGATGCAACGGTTTCGTTTCGTGGATCACGATGTTCAGGAGCAGCAAACTAGCGTCAGACGCTCGCAGGTCAGCGAACGAGGTTAGCGTTACCAAATCCGTCTTCACTTGTCGTAGCTGAGCCGCCGCGTTAGTTGCAACCTTCCGCTGTATTACCGTGATGAGATTG 4590
ATCCTACGTTGCCAAAGCAAAGCACCTAGTGCTACAAGTCCTCGTCGTTTGATCGCAGTCTGCGAGCGTCCAGTCGCTTGCTCCAATCGCAATGGTTTAGGCAGAAGTGAACAGCATCGACTCGGCGGCGCAATCAACGTTGGAAGGCGACATAATGGCACTACTCTAAC
3´crt

GGACCAACTACGTTGCATCGGACGTCGCATCCTTCACGGAGGAGCTCGCTGCCAGGTTCTGGTACGAGGGCCACAGTGATTACGACTTCCAGGAAGGTGGGTTACGAGAGTTAATAGTGTTTGACCATCATTGCAGGCGGTCCTGAGAACCGTAATCCCAATGTTTTGTC 4760
CCTGGTTGATGCAACGTAGCCTGCAGCGTAGGAAGTGCCTCCTCGAGCGACGGTCCAAGACCATGCTCCCGGTGTCACTAATGCTGAAGGTCCTTCCACCCAATGCTCTCAATTATCACAAACTGGTAGTAACGTCCGCCAGGACTCTTGGCATTAGGGTTACAAAACAG
3´crt

GTTTACGCAGCTCGTTTGGAAGTCCTCAAAGGTACCTTAGCACTCCCTGTTACATATTATATATGCCACAGGATGTTGGTTGCGGAGTTGCTTGCTGCGATAAGGAGCGGTTGATCCTTGTCTGTCGTTTTCACCCAGCTGGAAACATACAGGGTATGCTATGCTAGTGT 4930
CAAATGCGTCGAGCAAACCTTCAGGAGTTTCCATGGAATCGTGAGGGACAATGTATAATATATACGGTGTCCTACAACCAACGCCTCAACGAACGACGCTATTCCTCGCCAACTAGGAACAGACAGCAAAAGTGGGTCGACCTTTGTATGTCCCATACGATACGATCACA
3´crt

XbaI
GTCTAATGACATATTTATAGGCTACTATACACAGAACGTCCTTGAGAAGGTAAGTCACACTCCCTTAGTCTAGACtatagtgagtcgtattacaattcactggccgtcgttttacaacgtcgtgactgggaaaaccctggcgttacccaacttaatcgccttgcagcaca 5100
CAGATTACTGTATAAATATCCGATGATATGTGTCTTGCAGGAACTCTTCCATTCAGTGTGAGGGAATCAGATCTGatatcactcagcataatgttaagtgaccggcagcaaaatgttgcagcactgacccttttgggaccgcaatgggttgaattagcggaacgtcgtgt
3´crt | backbone
M13 fwd

tcccccttttcgccagctggcgtaatagcgaagaggcccgcaccgatcgcccttcccaacagttgcgcagcctgaatggcgaatggcgcctgatgcggtattttctccttacgcatctgtgcggtatttcacaccgcatatggtgcactctcagtacaatctgctctgatg 5270
agggggaaagcggtcgaccgcattatcgcttctccgggcgtggctagcgggaagggttgtcaacgcgtcggacttaccgcttaccgcggactacgccataaaagaggaatgcgtagacacgccataaagtgtggcgtataccacgtgagagtcatgttagacgagactac
backbone

ccgcatagttaagccagccccgacacccgccaacacccgctgacgcgccctgacgggcttgtctgctcccggcatccgcttacagacaagctgtgaccgtctccgggagctgcatgtgtcagaggttttcaccgtcatcaccgaaacgcgcgagacgaaagggcctcgtg 5440
ggcgtatcaattcggtcggggctgtgggcggttgtgggcgactgcgcgggactgcccgaacagacgagggccgtaggcgaatgtctgttcgacactggcagaggccctcgacgtacacagtctccaaaagtggcagtagtggctttgcgcgctctgctttcccggagcac
backbone

atacgcctatttttataggttaatgtcatgataataatggtttcttagacgtcaggtggcacttttcggggaaatgtgcgcggaacccctatttgtttatttttctaaatacattcaaatatgtatccgctcatgagacaataaccctgataaatgcttcaataatattg 5610
tatgcggataaaaatatccaattacagtactattattaccaaagaatctgcagtccaccgtgaaaagcccctttacacgcgccttggggataaacaaataaaaagatttatgtaagtttatacataggcgagtactctgttattgggactatttacgaagttattataac
backbone

AmpR promoter

aaaaaggaagagtatgagtattcaacatttccgtgtcgcccttattcccttttttgcggcattttgccttcctgtttttgctcacccagaaacgctggtgaaagtaaaagatgctgaagatcagttgggtgcacgagtgggttacatcgaactggatctcaacagcggta 5780
tttttccttctcatactcataagttgtaaaggcacagcgggaataagggaaaaaacgccgtaaaacggaaggacaaaaacgagtgggtctttgcgaccactttcattttctacgacttctagtcaaccacgtgctcacccaatgtagcttgacctagagttgtcgccat
backbone

1 5 10 15 20 25 30 35 40 45 50
Met Ser Ile Gln His Phe Arg Val Ala Leu Ile Pro Phe Phe Ala Ala Phe Cys Leu Pro Val Phe Ala His Pro Glu Thr Leu Val Lys Val Lys Asp Ala Glu Asp Gln Leu Gly Ala Arg Val Gly Tyr Ile Glu Leu Asp Leu Asn Ser Gly
signal sequence
AmpR promoter
AmpR
Met Ser Ile Gln His Phe Arg Val Ala Leu Ile Pro Phe Phe Ala Ala Phe Cys Leu Pro Val Phe Ala His Pro Glu Thr Leu Val Lys Val Lys Asp Ala Glu Asp Gln Leu Gly Ala Arg Val Gly Tyr Ile Glu Leu Asp Leu Asn Ser Gly

agatccttgagagttttcgccccgaagaacgttttccaatgatgagcacttttaaagttctgctatgtggcgcggtattatcccgtattgacgccgggcaagagcaactcggtcgccgcatacactattctcagaatgacttggttgagtactcaccagtcacagaaaag 5950
tctaggaactctcaaaagcggggcttcttgcaaaaggttactactcgtgaaaatttcaagacgatacaccgcgccataatagggcataactgcggcccgttctcgttgagccagcggcgtatgtgataagagtcttactgaaccaactcatgagtggtcagtgtcttttc
backbone

55 60 65 70 75 80 85 90 95 100 105
Lys Ile Leu Glu Ser Phe Arg Pro Glu Glu Arg Phe Pro Met Met Ser Thr Phe Lys Val Leu Leu Cys Gly Ala Val Leu Ser Arg Ile Asp Ala Gly Gln Glu Gln Leu Gly Arg Arg Ile His Tyr Ser Gln Asn Asp Leu Val Glu Tyr Ser Pro Val Thr Glu Lys
AmpR
Lys Ile Leu Glu Ser Phe Arg Pro Glu Glu Arg Phe Pro Met Met Ser Thr Phe Lys Val Leu Leu Cys Gly Ala Val Leu Ser Arg Ile Asp Ala Gly Gln Glu Gln Leu Gly Arg Arg Ile His Tyr Ser Gln Asn Asp Leu Val Glu Tyr Ser Pro Val Thr Glu Lys

catcttacggatggcatgacagtaagagaattatgcagtgctgccataaccatgagtgataacactgcggccaacttacttctgacaacgatcggaggaccgaaggagctaaccgcttttttgcacaacatgggggatcatgtaactcgccttgatcgttgggaaccgga 6120
gtagaatgcctaccgtactgtcattctcttaatacgtcacgacggtattggtactcactattgtgacgccggttgaatgaagactgttgctagcctcctggcttcctcgattggcgaaaaaacgtgttgtacccccctagtacattgagcggaactagcaacccttggcct
backbone

110 115 120 125 130 135 140 145 150 155 160 165
His Leu Thr Asp Gly Met Thr Val Arg Glu Leu Cys Ser Ala Ala Ile Thr Met Ser Asp Asn Thr Ala Ala Asn Leu Leu Leu Thr Thr Ile Gly Gly Pro Lys Glu Leu Thr Ala Phe Leu His Asn Met Gly Asp His Val Thr Arg Leu Asp Arg Trp Glu Pro Glu
AmpR
His Leu Thr Asp Gly Met Thr Val Arg Glu Leu Cys Ser Ala Ala Ile Thr Met Ser Asp Asn Thr Ala Ala Asn Leu Leu Leu Thr Thr Ile Gly Gly Pro Lys Glu Leu Thr Ala Phe Leu His Asn Met Gly Asp His Val Thr Arg Leu Asp Arg Trp Glu Pro Glu
Thr Val Arg Arg Ser Arg Gln Ser Gly Ser

gctgaatgaagccataccaaacgacgagcgtgacaccacgatgcctgtagcaatggcaacaacgttgcgcaaactattaactggcgaactacttactctagcttcccggcaacaattaatagactggatggaggcggataaagttgcaggaccacttctgcgctcggccc 6290
cgacttacttcggtatggtttgctgctcgcactgtggtgctacggacatcgttaccgttgttgcaacgcgtttgataattgaccgcttgatgaatgagatcgaagggccgttgttaattatctgacctacctccgcctatttcaacgtcctggtgaagacgcgagccggg
backbone

170 175 180 185 190 195 200 205 210 215 220
Leu Asn Glu Ala Ile Pro Asn Asp Glu Arg Asp Thr Thr Met Pro Val Ala Met Pro Thr Thr Leu Arg Lys Leu Leu Thr Gly Glu Leu Leu Thr Leu Ala Ser Arg Gln Gln Leu Ile Asp Trp Met Glu Ala Asp Lys Val Ala Gly Pro Leu Leu Arg Ser Ala
AmpR
Leu Asn Glu Ala Ile Pro Asn Asp Glu Arg Asp Thr Thr Met Pro Val Ala Met Pro Thr Thr Leu Arg Lys Leu Leu Thr Gly Glu Leu Leu Thr Leu Ala Ser Arg Gln Gln Leu Ile Asp Trp Met Glu Ala Asp Lys Val Ala Gly Pro Leu Leu Arg Ser Ala
Ser Phe Ser Ala Met Gly Phe Ser Ser Arg Ser Val Val Ile Gly Thr Ala Ile Gly Val Val Asn Arg Leu Ser Asn Val Pro Ser Ser Ser Val Arg Ala Glu Arg Cys Cys Asn Ile Ser Gln Ile Ser Ala Ser Leu Thr Ala Pro Gly Ser Arg Arg Glu Ala Arg

ttccggctggctggtttattgctgataaatctggagccggtgagcgtgggtctcgcggtatcattgcagcactggggccagatggtaagccctcccgtatcgtagttatctacacgacggggagtcaggcaactatggatgaacgaaatagacagatcgctgagataggt 6460
aaggccgaccgaccaaataacgactatttagacctcggccactcgcacccagagcgccatagtaacgtcgtgaccccggtctaccattcgggagggcatagcatcaatagatgtgctgcccctcagtccgttgatacctacttgctttatctgtctagcgactctatcca
backbone

225 230 235 240 245 250 255 260 265 270 275
Leu Pro Ala Gly Trp Phe Ile Ala Asp Lys Ser Gly Ala Gly Glu Arg Gly Ser Arg Gly Ile Ile Ala Ala Leu Gly Pro Asp Gly Lys Pro Ser Arg Ile Val Val Ile Tyr Thr Thr Gly Ser Gln Ala Thr Met Asp Glu Arg Asn Arg Gln Ile Ala Glu Ile Gly
AmpR
Leu Pro Ala Gly Trp Phe Ile Ala Asp Lys Ser Gly Ala Gly Glu Arg Gly Ser Arg Gly Ile Ile Ala Ala Leu Gly Pro Asp Gly Lys Pro Ser Arg Ile Val Val Ile Tyr Thr Thr Gly Ser Gln Ala Thr Met Asp Glu Arg Asn Arg Gln Ile Ala Glu Ile Gly
Gly Ala Pro Gln Asn Ile Ala Ser Leu Asp Pro Ala Pro Ser Arg Pro Asp Arg Pro Ile Met

gcctcactgattaagcattggtaactgtcagaccaagtttactcatatatactttagattgatttaaaacttcatttttaatttaaaaggatctaggtgaagatcctttttgataatctcatgaccaaaatcccttaacgtgagttttcgttccactgagcgtcagaccc 6630
cggagtgactaattcgtaaccattgacagtctggttcaaatgagtatatatgaaatctaactaaattttgaagtaaaaattaaattttcctagatccacttctaggaaaaactattagagtactggttttagggaattgcactcaaaagcaaggtgactcgcagtctggg
backbone

280 285
Ala Ser Leu Ile Lys His Trp
AmpR
Ala Ser Leu Ile Lys His Trp

cgtagaaaagatcaaaggatcttcttgagatcctttttttctgcgcgtaatctgctgcttgcaaacaaaaaaaccaccgctaccagcggtggtttgtttgccggatcaagagctaccaactctttttccgaaggtaactggcttcagcagagcgcagataccaaatactg 6800
gcatcttttctagtttcctagaagaactctaggaaaaaaagacgcgcattagacgacgaacgtttgtttttttggtggcgatggtcgccaccaaacaaacggcctagttctcgatggttgagaaaaaggcttccattgaccgaagtcgtctcgcgtctatggtttatgac
backbone

ori

tccttctagtgtagccgtagttaggccaccacttcaagaactctgtagcaccgcctacatacctcgctctgctaatcctgttaccagtggctgctgccagtggcgataagtcgtgtcttaccgggttggactcaagacgatagttaccggataaggcgcagcggtcgggc 6970
aggaagatcacatcggcatcaatccggtggtgaagttcttgagacatcgtggcggatgtatggagcgagacgattaggacaatggtcaccgacgacggtcaccgctattcagcacagaatggcccaacctgagttctgctatcaatggcctattccgcgtcgccagcccg
backbone

ori

tgaacggggggttcgtgcacacagcccagcttggagcgaacgacctacaccgaactgagatacctacagcgtgagctatgagaaagcgccacgcttcccgaagggagaaaggcggacaggtatccggtaagcggcagggtcggaacaggagagcgcacgagggagcttcc 7140
acttgccccccaagcacgtgtgtcgggtcgaacctcgcttgctggatgtggcttgactctatggatgtcgcactcgatactctttcgcggtgcgaagggcttccctctttccgcctgtccataggccattcgccgtcccagccttgtcctctcgcgtgctccctcgaagg
backbone

ori

aggggggaaacgcctggtatctttatagtcctgtcgggtttcgccacctctgacttgagcgtcgatttttgtgatgctcgtcaggggggcggagcctatcgaaaaacgccagcaacgcggcctttttacggttcctggccttttgctggccttttgctcacatgttctttc 7310
tccccctttgcggaccatagaaatatcaggacagcccaaagcggtggagactgaactcgcagctaaaaacactacgagcagtccccccgcctcggatagctttttgcggtcgttgcgccggaaaaatgccaaggaccggaaaacgaccggaaaacgagtgtacaagaaag

backbone

ori

ctgcgttatcccctgattctgtggataaccgtattaccgcctttgagtgagctgataccgctcgccgcagccgaacgaccgagcgcagcgagtcagtgagcgaggaagcggaagagcgcccaatacgcaaaccgcctctccccgcgcgttggccgattcattaatgcagc 7480
gacgcaataggggactaagacacctattggcataatggcggaaactcactcgactatggcgagcggcgtcggcttgctggctcgcgtcgctcagtcactcgctccttcgccttctcgcgggttatgcgtttggcggagaggggcgcgcaaccggctaagtaattacgtcg

backbone

tggcacgacaggtttcccgactggaaagcgggcagtgagcgcaacgcaattaatgtgagttagctcactcattaggcaccccaggctttacactttatgcttccggctcgtatgttgtgtggaattgtgagcggataacaatttcacacaggaaacagctatgaccatga 7650
accgtgctgtccaaagggctgacctttcgcccgtcactcgcgttgcgttaattacactcaatcgagtgagtaatccgtggggtccgaaatgtgaaatacgaaggccgagcatacaacacaccttaacactcgcctattgttaaagtgtgtcctttgtcgatactggtact

backbone

CAP binding site | -35 | -10 | lac promoter | lac operator | M13 rev

XhoI

ttacgccaagctatttaggtgacactatagaatactcCTCGAG 3′
7693
aatgcggttcgataaatccactgtgatatcttatgagGAGCTC 5′

backbone

SP6 promoter

Alias: 6-cys-e-gfp-ef-tgtp-hdhfr

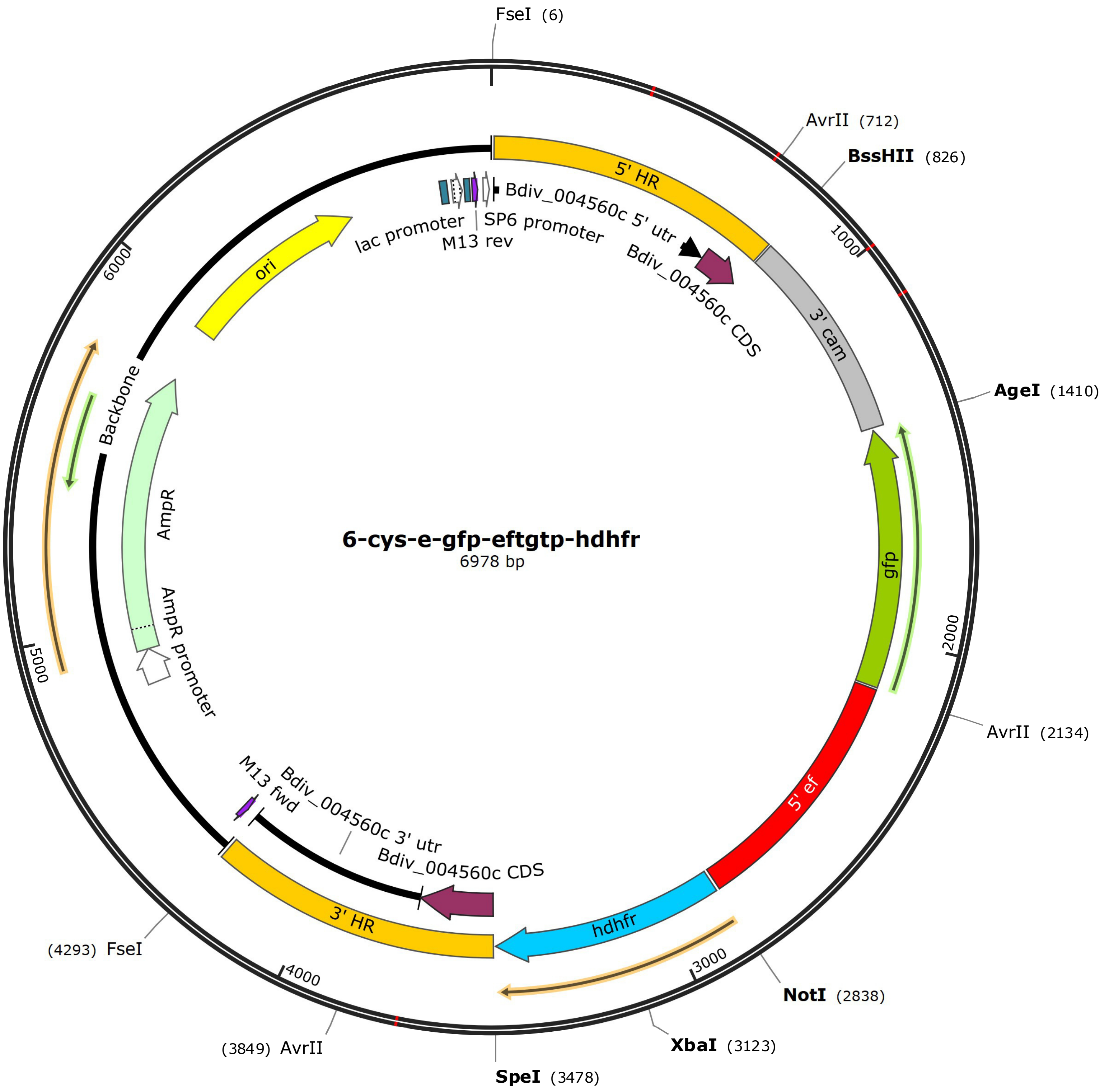

Sequence: 3. 6-cys-e-gfp-ef-tgtp-hdhfr.dna (Circular / 6978 bp)
Enzymes: < Unsaved Enzyme Set > (7 of 678 total)
Features: 20 total

Unique Cutters **Bold**

FseI

5′ GGCCGGCCCAAACTGCCGCATTCACACCACTCGAGCACTACTCGTGCATGCTGGTATCACTAATGCCATCACTCACCCATATCTATCTCGTCGTTAATTGTTGCTTTGTCTGCACTGTCGGGGTTGAAGCCGTTTAGGTGAATGCAGCGCACGCAACGGTCATGCTGCAT 170
3′ CCGGCCGGGTTTGACGGCGTAAGTGTGGTGAGCTCGTGATGAGCACGTACGACCATAGTGATTACGGTAGTGAGTGGGTATAGATAGAGCAGCAATTAACAACGAAACAGACGTGACAGCCCCAACTTCGGCAAATCCACTTACGTCGCGTGCGTTGCCAGTACGACGTA

5' HR

Bdiv_004560c 5' utr

CATGAGCTGTATCTTCACGGTGCGCACAAAACTCGTTGAGCCAATCCTTGCGGGTTAGGCACCCATCTATGTTCGTATACTCCTTGAGATTCTCGAACTCCTGCTTGTAGAAGCGCTTCACAACATAGTGATTGCCCACGGAATCACATGAGAGGTACACATCTTCATACT 340
GTACTCGACATAGAAGTGCCACGCGTGTTTTGAGCAACTCGGTTAGGAACGCCCAATCCGTGGGTAGATACAAGCATATGAGGAACTCTAAGAGCTTGAGGACGAACATCTTCGCGAAGTGTTGTATCACTAACGGGTGCCTTAGTGTACTCTCCATGTGTAGAAGTATGA

5' HR

Bdiv_004560c 5' utr

TCCCATAGTGTAGGAGCTTTATGAGAGACAGCTCGTTCtTTTAATACAAGAGATGAGATAGCAAACTTTGTTCAATGATTTATCACCGTAGAAGGCAGACGATGTCGGGAATTCATTTCGCCACGTCTGACCTTCTTAAGGCGATACACACCTGTGGGCACAAAATGTGA 510
AGGGTATCACATCCTCGAAATACTCTCTGTCGAGCAAGaAAATTATGTTCTCTACTCTATCGTTTGAAACAAGTTACTAAATAGTGGCATCTTCCGTCTGCTACAGCCCTTAAGTAAAGCGGTGCAGACTGGAAGAATTCCGCTATGTGTGGACACCCGTGTTTTACACT

5' HR

Bdiv_004560c 5' utr

GTCTTTTGACTCATATACACTTTCCAATTGCACCTGTGTACGTTGAGTGTGCCATGATGACACTTAAACAAGTGACACAACTAATTACTTTATCCAGGGTACGGATAAAAGAAGGGTTTTACGAAGCTGCTGGACTTAAACAATCAGCATCATGGCGTTCCAAAATACAC 680
CAGAAAACTGAGTATATGTGAAAGGTTAACGTGGACACATGCAACTCACACGGTACTACTGTGAATTTGTTCACTGTGTTGATTAATGAAATAGGTCCCATGCCTATTTTCTTCCCAAAATGCTTCGACGACCTGAATTTGTTAGTCGTAGTACCGCAAGGTTTTATGTG

5' HR

Bdiv_004560c 5' utr

AvrII **BssHII**

GATGGAGTGTCTTTATCACCATGATGCTCGCCCTAGGCGTGCTCAATCCGGGTGTACAGGCGCTTGGTGACAGTTGGTCCCTCAAAGATATATTCAAGAAGTCCGTAAACCACACCACCAGCTATGCCTTGTTGGCAAACACCATGCGCGCATTGCTGTGTACTGGACCG 850
CTACCTCACAGAAATAGTGGTACTACGAGCGGGATCCGCACGAGTTAGGCCCACATGTCCGCGAACCACTGTCAACCAGGGAGTTTCTATATAAGTTCTTCAGGCATTTGGTGTGGTGGTCGATACGGAACAACCGTTTGTGGTACGCGCGTAACGACACATGACCTGGC

5' HR | 3' cam

Bdiv_004560c 5' utr | Bdiv_004560c CDS

ACGCGTTGGCCTCTGTAGCCTTGGTCTTCTGGTCATCATTTTCCCCTGTTCGCGCTGATACTTTGCGTAATACGGCTTACCCTCTCGTTTCTCTCGCGCATAGTAGTCGCATAGCGATTTGATATAGTTGTTGATGTTCAAAACCTTATGCtTCACGCTCTCCTTGATGG 1020
TGCGCAACCGGAGACATCGGAACCAGAAGACCAGTAGTAAAAGGGGACAAGCGCGACTATGAAACGCATTATGCCGAATGGGAGAGCAAAGAGAGCGCGTATCATCAGCGTATCGCTAAACTATATCAACAACTACAAGTTTTGGAATACGaAGTGCGAGAGGAACTACC

3' cam

AGGTGAGATCGCATCCTCGGTTGGCTTCAGTTAGGTACTCATCCGCGACCTTAACAAGTCCTATGAGCAGGGTGTACTTCCCACACTCGGTTTTTGCCGGTTCAGGCTCCtTTTTGCGCGATGTGTGCCCGTAAGACAGGCAGCTAATGTACAGGTGTGTAGGCGCCGAT 1190
TCCACTCTAGCGTAGGAGCCAACCGAAGTCAATCCATGAGTAGGCGCTGGAATTGTTCAGGATACTCGTCCCACATGAAGGGTGTGAGCCAAAAACGGCCAAGTCCGAGGaAAAACGCGCTACACACGGGCATTCTGTCCGTCGATTACATGTCCACACATCCGCGGCTA

3' cam

GCGTGGGAGCTCAGCGCATCGCCACGTGTATTTGATCACGCGGCTTAATTTATGCTTAACCATCACGCCTAGCGACGGCGAAAACGCCGACCAAGTGTTTATTTATGGGGTCTGCCTTTGGCGCTGGTTGATAGGATTAAGGGTACATTTACTACGGTGAGATAGTTGTG 1360
CGCACCCTCGAGTCGCGTAGCGGTGCACATAAACTAGTGCGCCGAATTAAATACGAATTGGTAGTGCGGATCGCTGCCGCTTTTGCGGCTGGTTCACAAATAAATACCCCAGACGGAAACCGCGACCAACTATCCTAATTCCCATGTAAATGATGCCACTCTATCAACAC

3' cam

AgeI

TAACCAAATACTATTTGATTTCGTATAATGTGTTCGAACCGGCATCTGAACCGGTCTTATTTATAAAGCTCATCCATTCCGTGAGTAATACCGGCGGCGGTCACGAATTCTAATAAAACCATGTGGTCACGTTTCTCGTTTGGATCCTTACTTAATTTACTTTGTGTAGA 1530
ATTGGTTTATGATAAACTAAAGCATATTACACAAGCTTGGCCGTAGACTTGGCCAGAATAAATATTTCGAGTAGGTAAGGCACTCATTATGGCCGCCGCCAGTGCTTAAGATTATTTTGGTACACCAGTGCAAAGAGCAAACCTAGGAATGAATTAAATGAAACACATCT

3' cam | gfp

Lys Tyr Leu Glu Asp Met Gly His Thr Ile Gly Ala Ala Thr Val Phe Glu Leu Leu Val Met His Asp Arg Lys Glu Asn Pro Asp Lys Ser Leu Lys Ser Gln Thr Ser

AAGATAGTGATTGTCAGGCAACAATACAGGACCATCACCAATAGGTGTATTTTGCTGATAGTGGTCAGCTAATTGCACACTTCCATCCTCAATGTTATGCCTAATCTTAAAGTTTACTTTTATACCATTTTTCTGCTTGTCTGCCATAATATACACATTATGACTGTTGT 1700
TTCTATCACTAACAGTCCGTTGTTATGTCCTGGTAGTGGTTATCCACATAAAACGACTATCACCAGTCGATTAACGTGTGAAGGTAGGAGTTACAATACGGATTAGAATTTCAAATGAAAATATGGTAAAAAGACGAACAGACGGTATTATATGTGTAATACTGACAACA

gfp

Leu Tyr His Asn Asp Pro Leu Leu Val Pro Gly Asp Gly Ile Pro Thr Asn Gln Gln Tyr His Asp Ala Leu Gln Val Ser Gly Asp Glu Ile Asn His Arg Ile Lys Phe Asn Val Lys Ile Gly Asn Lys Gln Lys Asp Ala Met Ile Tyr Val Asn His Ser Asn Tyr

AATTGTATTCCAACTTATGTCCTAAAATATTACCATCCTCCTTAAAATCAATACCTTTAAGTTCTATCCTGTTAACAAGGGTGTCACCCTCAAATTTTACCTCTGCACGAGTTTTGTAGTTACCGTCGTCTTTGAAGAAAATTGTTCTCTCCTGAACGTATCCCTCTGG 1870
TTAACATAAGGTTGAATACAGGATTTTATAATGGTAGGAGGAATTTTAGTTATGGAAATTCAAGATAGGACAATTGTTCCCACAGTGGGAGTTTAAAATGGAGACGTGCTCAAAACATCAATGGCAGCAGAAACTTCTTTTAACAAGAGAGGACTTGCATAGGGAGACC

gfp

Asn Tyr Glu Leu Lys His Gly Leu Ile Asn Gly Asp Glu Lys Phe Asp Ile Gly Lys Leu Glu Ile Arg Asn Val Leu Thr Asp Gly Glu Phe Lys Val Glu Ala Arg Thr Lys Tyr Asn Gly Asp Asp Lys Phe Phe Ile Thr Arg Glu Gln Val Tyr Gly Glu Pro

ATAGCAGACTTAAAGAAGTCGTGTTGCTTCATGTGATCTGGGTAACGTGAGAAGCACTGAACTCCATAAGTCAAGGTGGTAACTAAAGTAGGCCATGGTACTGGAAGTTTTCCTGTAGTGCAAATGAACTTTAAAGTTAATTTACCGTATGTAGCATCACCTTCTCCTTC 2040
TATCGTCTGAATTTCTTCAGCACAACGAAGTACACTAGACCCATTGCACTCTTCGTGACTTGAGGTATTCAGTTCCACCATTGATTTCATCCGGTACCATGACCTTCAAAAGGACATCACGTTTACTTGAAATTTCAATTAAATGGCATACATCGTAGTGGAAGAGGAAG

gfp

Met Ala Ser Lys Phe Phe Asp His Gln Lys Met His Asp Pro Tyr Arg Ser Phe Cys Gln Val Gly Tyr Thr Leu Thr Thr Val Leu Thr Pro Trp Pro Val Pro Leu Lys Gly Thr Thr Cys Ile Phe Lys Leu Thr Leu Lys Gly Tyr Thr Ala Asp Gly Glu Gly Glu

AvrII

ACCAGAAACTGAAAATTTGTGACCATTTACATCTCCGTCCAATTTCTACAAGAATTGGAACAACTCCAGTAAACAACTCTTCTCCCTTACTCATCCTAGGGTTTGGCACTGTTGCTCCGCCCAGGTGAAGTTACGTAGTCTTATAATGCTGATCAAAGGAACACTTGTTGA 2210
TGGTCTTTGACTTTTAAACACTGGTAAATGTAGAGGCAGGTTAAGATGTTCTTAACCTTGTTGAGGTCATTTGTTGAGAAGAGGGAATGAGTAGGATCCCAAACCGTGACAACGAGGCGGGTCCACTTCAATGCATCAGAATATTACGACTAGTTTCCTTGTGAACAACT

gfp | 5' ef

Gly Ser Val Ser Phe Lys His Gly Asn Val Asp Gly Asp Leu Glu Val Leu Ile Pro Val Val Gly Thr Phe Leu Glu Glu Gly Lys Ser Met

AGCTCCTGTGCCCCGTCATATTCTTTTCACGGGTGTTATTTTTAGCGAATTAGTTTATTTACCTGTATGGGTGGTACTGCGCCTTGCGGTTCCCAACAAGCGTCTCTATTATAGTGGCATGACTACCACGATTAGTTTAGATTCTGCGATTATTTCATCGAGACATGTGT 2380
TCGAGGACACGGGGCAGTATAAGAAAAGTGCCCACAATAAAAATCGCTTAATCAAATAAATGGACATACCCACCATGACGCGGAACGCCAAGGGTTGTTCGCAGAGATAATATCACCGTACTGATGGTGCTAATCAAATCTAAGACGCTAATAAAGTAGCTCTGTACACA

5' ef

ATGTCGAGTAGGTGTTTCATATCTTTCGTTTTATCGTTGCGTTTATAGTCACGCGTTACTCCTCTCTGTGCGAGTTCGTTCATTGTATGCTATCCGTCACGGCGTATAACTAGAACGTGCACATAGTGTTTATATATATTATTTATGTACATTCGTATTAGGGCGCAATA 2550
TACAGCTCATCCACAAAGTATAGAAAGCAAAATAGCAACGCAAATATCAGTGCGCAATGAGGAGAGACACGCTCAAGCAAGTAACATACGATAGGCAGTGCCGCATATTGATCTTGCACGTGTATCACAAATATATATAATAAATACATGTAAGCATAATCCCGCGTTAT

5' ef

TTTGTCTCCTGGCGTGGGTGTTTGTTTCTCTTGGCTGGAGGACAGCTCTGTGCCTTGATGTTATACGTCTGCCTTATATATGCGCCGCGGTTCGATGTTATGTGAGATATAGTTCCATATTAACGATACAATAGCCTATATCGCCATTACATTTTCCTACTCCGGTATAA 2720
AAACAGAGGACCGCACCCACAAACAAAGAGAACCGACCTCCTGTCGAGACACGGAACTACAATATGCAGACGGAATATATACGCGGCGCCAAGCTACAATACACTCTATATCAAGGTATAATTGCTATGTTATCGGATATAGCGGTAATGTAAAAGGATGAGGCCATATT
5' ef

NotI
TTATACTGTAATATGCGTTCTTCACGTTTACATCTTGTGTACTGTAATAAACCTCGCATTTGCATGTATACAGCTGATATTATAATCCTTTGCAGATTAATGGTACCTTGTAATCTGCGGCCGCATGGTTGGTTCGCTAAACTGCATCGTCGCTGTGTCCCAGAACATGG 2890
AATATGACATTATACGCAAGAAGTGCAAATGTAGAACACATGACATTATTTGGAGCGTAAACGTACATATGTCGACTATAATATTAGGAAACGTCTAATTACCATGGAACATTAGACGCCGGCGTACCAACCAAGCGATTTGACGTAGCAGCGACACAGGGTCTTGTACC
1 5 10 15
Met Val Gly Ser Leu Asn Cys Ile Val Ala Val Ser Gln Asn Met
5' ef
hdhfr
Met Val Gly Ser Leu Asn Cys Ile Val Ala Val Ser Gln Asn Met

GCATCGGCAAGAACGGGGACCTGCCCTGGCCACCGCTCAGGAACGAATTTAGATATTTCCAGAGAATGACCACAACCTCTTCAGTAGAAGGTAAACAGAATCTGGTGATTATGGGTAAGAAGACCTGGTTCTCCATTCCTGAGAAGAATCGACCTTTAAAGGGTAGAATT 3060
CGTAGCCGTTCTTGCCCCTGGACGGGACCGGTGGCGAGTCCTTGCTTAAATCTATAAAGGTCTCTTACTGGTGTTGGAGAAGTCATCTTCCATTTGTCTTAGACCACTAATACCCATTCTTCTGGACCAAGAGGTAAGGACTCTTCTTAGCTGGAAATTTCCCATCTTAA
20 25 30 35 40 45 50 55 60 65 70
Gly Ile Gly Lys Asn Gly Asp Leu Pro Trp Pro Pro Leu Arg Asn Glu Phe Arg Tyr Phe Gln Arg Met Thr Thr Thr Ser Ser Val Glu Gly Lys Gln Asn Leu Val Ile Met Gly Lys Lys Thr Trp Phe Ser Ile Pro Glu Lys Asn Arg Pro Leu Lys Gly Arg Ile
hdhfr
Gly Ile Gly Lys Asn Gly Asp Leu Pro Trp Pro Pro Leu Arg Asn Glu Phe Arg Tyr Phe Gln Arg Met Thr Thr Thr Ser Ser Val Glu Gly Lys Gln Asn Leu Val Ile Met Gly Lys Lys Thr Trp Phe Ser Ile Pro Glu Lys Asn Arg Pro Leu Lys Gly Arg Ile

XbaI
AATTTAGTTCTCAGCAGAGAACTCAAGGAACCTCCACAAGGAGCTCATTTTCTTTCCAGAAGTCTAGATGATGCCTTAAAACTTACTGAACAACCAGAATTAGCAAATAAAGTAGACATGGTCTGGATAGTTGGTGGCAGTTCTGTTTATAAGGAAGCCATGAATCACC 3230
TTAAATCAAGAGTCGTCTCTTGAGTTCCTTGGAGGTGTTCCTCGAGTAAAAGAAAGGTCTTCAGATCTACTACGGAATTTTGAATGACTTGTTGGTCTTAATCGTTTATTTCATCTGTACCAGACCTATCAACCACCGTCAAGACAAATATTCCTTCGGTACTTAGTGG
75 80 85 90 95 100 105 110 115 120 125
Asn Leu Val Leu Ser Arg Glu Leu Lys Glu Pro Pro Gln Gly Ala His Phe Leu Ser Arg Ser Leu Asp Asp Ala Leu Lys Leu Thr Glu Gln Pro Glu Leu Ala Asn Lys Val Asp Met Val Trp Ile Val Gly Gly Ser Ser Val Tyr Lys Glu Ala Met Asn His Pro
hdhfr
Asn Leu Val Leu Ser Arg Glu Leu Lys Glu Pro Pro Gln Gly Ala His Phe Leu Ser Arg Ser Leu Asp Asp Ala Leu Lys Leu Thr Glu Gln Pro Glu Leu Ala Asn Lys Val Asp Met Val Trp Ile Val Gly Gly Ser Ser Val Tyr Lys Glu Ala Met Asn His Pro

AGGCCATCTTAAACTATTTGTGACAAGGATCATGCAAGACTTTGAAAGTGACACGTTTTTTCCAGAAATTGATTTGGAGAAATATAAACTTCTGCCAGAATACCCAGGTGTTCTCTCTGATGTCCAGGAGGAGAAAGGCATTAAGTACAAATTTGAAGTATATGAGAAGA 3400
TCCGGTAGAATTTGATAAACACTGTTCCTAGTACGTTCTGAAACTTTCACTGTGCAAAAAAGGTCTTTAACTAAACCTCTTTATATTTGAAGACGGTCTTATGGGTCCACAAGAGAGACTACAGGTCCTCCTCTTTCCGTAATTCATGTTTAAACTTCATATACTCTTCT
130 135 140 145 150 155 160 165 170 175 180 185
Gly His Leu Lys Leu Phe Val Thr Arg Ile Met Gln Asp Phe Glu Ser Asp Thr Phe Phe Pro Glu Ile Asp Leu Glu Lys Tyr Lys Leu Leu Pro Glu Tyr Pro Gly Val Leu Ser Asp Val Gln Glu Glu Lys Gly Ile Lys Tyr Lys Phe Glu Val Tyr Glu Lys
hdhfr
Gly His Leu Lys Leu Phe Val Thr Arg Ile Met Gln Asp Phe Glu Ser Asp Thr Phe Phe Pro Glu Ile Asp Leu Glu Lys Tyr Lys Leu Leu Pro Glu Tyr Pro Gly Val Leu Ser Asp Val Gln Glu Glu Lys Gly Ile Lys Tyr Lys Phe Glu Val Tyr Glu Lys

SpeI
ATGATGCTATCAGATGCCGGTTCGAACACATTATACGAAATCAAATAGTATTTGGTTACACAACTATCTCACCGTAGACTAGTCTATTTTGGTTTCTACAGGTAGAAAGGTTGCTGTGAGTGACTATGTCCCTCTAGCCAGGGTTGTCAAGTCCAAGAACATAAAGGTCC 3570
TACTACGATAGTCTACGGCCAAGCTTGTGTAATATGCTTTAGTTTATCATAAACCAATGTGTTGATAGAGTGGCATCTGATCAGATAAAACCAAAGATGTCCATCTTTCCAACGACACTCACTGATACAGGGAGATCGGTCCCAACAGTTCAGGTTCTTGTATTTCCAGG
190 195 200 205 210
Asn Asp Ala Ile Arg Cys Arg Phe Glu His Ile Ile Arg Asn Gln Ile Val Phe Gly Tyr Thr Thr Ile Ser Pro *
hdhfr
3' HR
Bdiv_004560c CDS
Asn Asp Ala Ile Arg Cys Arg Phe Glu His Ile Ile Arg Asn Gln Ile Val Phe Gly Tyr Thr Thr Ile Ser Pro *

TTGACTTCTCTATCCCATCACGTCTGAAGACTGGTGTCACATATTCCAATGAAAAGCTACAGTGCAAGTGCCAGCGCAATGATGGAGCAGTTATGGCGACGATCACACTGGACTTGAGCAATCCCTATCCGCGAGCATGAGAGGCCGATCCAATGGTGGCAAAATCGTCC 3740
AACTGAAGAGATAGGGTAGTGCAGACTTCTGACCACAGTGTATAAGGTTACTTTTCGATGTCACGTTCACGGTCGCGTTACTACCTCGTCAATACCGCTGCTAGTGTGACCTGAACTCGTTAGGGATAGGCGCTCGTACTCTCCGGCTAGGTTACCACCGTTTTAGCAGG
3' HR
Bdiv_004560c CDS
Bdiv_004560c 3' utr

AvrII
TCCGCAATAGGTAGCAATTACCTGGTTCACCTTAAATTGGCGATTAACATCAAGCATTATGTGCATTGTAACATTATGTATAAAATTGTGAGTTAGGGTTTCAGTCGTCCTAGGGCTGCCTTAGATGAGAATGCAGTAATGGTTTATTACGCCTGTGAAGCTTAGTTATC 3910
AGGCGTTATCCATCGTTAATGGACCAAGTGGAATTTAACCGCTAATTGTAGTTCGTAATACACGTAACATTGTAATACATATTTTAACACTCAATCCCAAAGTCAGCAGGATCCCGACGGAATCTACTCTTACGTCATTACCAAATAATGCGGACACTTCGAATCAATAG
3' HR
Bdiv_004560c 3' utr

TATTTATGAGTACAATTATGTTGATTGCCTACTCCACACATATGATAAGGGCAGTCTGTGCCAATCTGGGTCTAATTCTATCTCCTCAGTAGATAAGACAGCACAACATGTTAGGTGTATACATTCGCTTACGGCGACCACCCACTTTAGTTTAAAGTTTTTGCATGGCT 4080
ATAAATACTCATGTTAATACAACTAACGGATGAGGTGTGTATACTATTCCCGTCAGACACGGTTAGACCCAGATTAAGATAGAGGAGTCATCTATTCTGTCGTGTTGTACAATCCACATATGTAAGCGAATGCCGCTGGTGGGTGAAATCAAATTTCAAAAACGTACCGA
3' HR
Bdiv_004560c 3' utr

TGACAGAGATTATAGAACAAACACTACATGTGCAGATGATAGTCACGTTTACTCACCAGTGTCACTGGGCTGGGCCTGTGCTCGTCAGACTGACTCATGTCACGTGCCAGAGAGCCCAATCTTGTATGTATTTACTATCCCAATGTTCTAGGGGTGCCAGATGTATGTCA 4250
ACTGTCTCTAATATCTTGTTTGTGATGTACACGTCTACTATCAGTGCAAATGAGTGGTCACAGTGACCCGACCCGGACACGAGCAGTCTGACTGAGTACAGTGCACGGTCTCTCGGGTTAGAACATACATAAATGATAGGGTTACAAGATCCCCACGGTCTACATACAGT
3' HR
Bdiv_004560c 3' utr

FseI
CGCAAAATGAAAAACTTTCAACGGAGAATTCAGCTTAGGCCGGCCctatagtgagtcgtattacaattcactggccgtcgttttacaacgtcgtgactgggaaaaccctggcgttacccaacttaatcgccttgcagcacatccccctttcgccagctggcgtaatagcg 4420
GCGTTTTACTTTTTGAAAGTTGCCTCTTAAGTCGAATCCGGCCGGgatatcactcagcataatgttaagtgaccggcagcaaaatgttgcagcactgacccttttgggaccgcaatgggttgaattagcggaacgtcgtgtagggggaaagcggtcgaccgcattatcgc
3' HR
Backbone
Bdiv_004560c 3' utr
M13 fwd

aagaggcccgcaccgatcgcccttcccaacagttgcgcagcctgaatggcgaatggcgcctgatgcggtattttctccttacgcatctgtgcggtatttcacaccgcatatggtgcactctcagtacaatctgctctgatgccgcatagttaagccagccccgacacccg 4590
ttctccgggcgtggctagcgggaagggttgtcaacgcgtcggacttaccgcttaccgcggactacgccataaaagaggaatgcgtagacacgccataaagtgtggcgtataccacgtgagagtcatgttagacgagactacggcgtatcaattcggtcggggctgtgggc
Backbone

ccaacacccgctgacgcgccctgacgggcttgtctgctcccggcatccgcttacagacaagctgtgaccgtctccgggagctgcatgtgtcagaggttttcaccgtcatcaccgaaacgcgcgagacgaaagggcctcgtgatacgcctatttttataggttaatgtcat 4760
ggttgtgggcgactgcgcgggactgcccgaacagacgagggccgtaggcgaatgtctgttcgacactggcagaggccctcgacgtacacagtctccaaaagtggcagtagtggctttgcgcgctctgctttcccggagcactatgcggataaaaatatccaattacagta
Backbone

gataataatggtttcttagacgtcaggtggcacttttcggggaaatgtgcgcggaacccctatttgtttatttttctaaatacattcaaatatgtatccgctcatgagacaataaccctgataaatgcttcaataatattgaaaaaggaagagtatgagtattcaacatt 4930
ctattattaccaaagaatctgcagtccaccgtgaaaagcccctttacacgcgccttggggataaacaaataaaaagatttatgtaagtttatacataggcgagtactctgttattgggactatttacgaagttattataactttttccttctcatactcataagttgtaa

Backbone

AmpR promoter

1 5
Met Ser Ile Gln His
signal sequence
AmpR

Met Ser Ile Gln His

tccgtgtcgcccttattcccttttttgcggcattttgccttcctgtttttgctcacccagaaacgctggtgaaagtaaaagatgctgaagatcagttgggtgcacgagtgggttacatcgaactggatctcaacagcggtaagatccttgagagttttcgccccgaagaa 5100
aggcacagcgggaataagggaaaaaacgccgtaaaacggaaggacaaaaacgagtgggtctttgcgaccactttcattttctacgacttctagtcaacccacgtgctcacccaatgtagcttgacctagagttgtcgccattctaggaactctcaaaagcggggcttctt

Backbone

10 15 20 25 30 35 40 45 50 55 60
Phe Arg Val Ala Leu Ile Pro Phe Phe Ala Ala Phe Cys Leu Pro Val Phe Ala His Pro Glu Thr Leu Val Lys Val Lys Asp Ala Glu Asp Gln Leu Gly Ala Arg Val Gly Tyr Ile Glu Leu Asp Leu Asn Ser Gly Lys Ile Leu Glu Ser Phe Arg Pro Glu Glu
signal sequence
AmpR

Phe Arg Val Ala Leu Ile Pro Phe Phe Ala Ala Phe Cys Leu Pro Val Phe Ala His Pro Glu Thr Leu Val Lys Val Lys Asp Ala Glu Asp Gln Leu Gly Ala Arg Val Gly Tyr Ile Glu Leu Asp Leu Asn Ser Gly Lys Ile Leu Glu Ser Phe Arg Pro Glu Glu

cgttttccaatgatgagcacttttaaagttctgctatgtggcgcggtattatcccgtattgacgccgggcaagagcaactcggtcgccgcatacactattctcagaatgacttggttgagtactcaccagtcacagaaaagcatcttacggatggcatgacagtaagaga 5270
gcaaaaggttactactcgtgaaaatttcaagacgatacaccgcgccataatagggcataactgcggcccgttctcgttgagccagcggcgtatgtgataagagtcttactgaaccaactcatgagtggtcagtgtcttttcgtagaatgcctaccgtactgtcattctct

Backbone

65 70 75 80 85 90 95 100 105 110 115
Arg Phe Pro Met Met Ser Thr Phe Lys Val Leu Leu Cys Gly Ala Val Leu Ser Arg Ile Asp Ala Gly Gln Glu Gln Leu Gly Arg Arg Ile His Tyr Ser Gln Asn Asp Leu Val Glu Tyr Ser Pro Val Thr Glu Lys His Leu Thr Asp Gly Met Thr Val Arg Glu
AmpR

Arg Phe Pro Met Met Ser Thr Phe Lys Val Leu Leu Cys Gly Ala Val Leu Ser Arg Ile Asp Ala Gly Gln Glu Gln Leu Gly Arg Arg Ile His Tyr Ser Gln Asn Asp Leu Val Glu Tyr Ser Pro Val Thr Glu Lys His Leu Thr Asp Gly Met Thr Val Arg Glu

attatgcagtgctgccataaccatgagtgataacactgcggccaacttacttctgacaacgatcggaggaccgaaggagctaaccgcttttttgcacaacatgggggatcatgtaactcgccttgatcgttgggaaccggagctgaatgaagccataccaaacgacgagc 5440
taatacgtcacgacggtattggtactcactattgtgacgccggttgaatgaagactgttgctagcctcctggcttcctcgattggcgaaaaaacgtgttgtaccccctagtacattgagcggaactagcaacccttggcctcgacttacttcggtatggtttgctgctcg

Backbone

120 125 130 135 140 145 150 155 160 165 170 175
Leu Cys Ser Ala Ala Ile Thr Met Ser Asp Asn Thr Ala Ala Asn Leu Leu Leu Thr Thr Ile Gly Gly Pro Lys Glu Leu Thr Ala Phe Leu His Asn Met Gly Asp His Val Thr Arg Leu Asp Arg Trp Glu Pro Glu Leu Asn Glu Ala Ile Pro Asn Asp Glu
AmpR

Leu Cys Ser Ala Ala Ile Thr Met Ser Asp Asn Thr Ala Ala Asn Leu Leu Leu Thr Thr Ile Gly Gly Pro Lys Glu Leu Thr Ala Phe Leu His Asn Met Gly Asp His Val Thr Arg Leu Asp Arg Trp Glu Pro Glu Leu Asn Glu Ala Ile Pro Asn Asp Glu

* Thr Val Arg Arg Ser Arg Gln Ser Gly Ser Ser Phe Ser Ala Met Gly Phe Ser Ser Arg

gtgacaccacgatgcctgtagcaatgccaacaacgttgcgcaaactattaactggcgaactacttactctagcttcccggcaacaattaatagactggatggaggcggataaagttgcaggaccacttctgcgctcggcccttccggctggctggtttattgctgataaa 5610
cactgtggtgctacggacatcgttacggttgttgcaacgcgtttgataattgaccgcttgatgaatgagatcgaagggccgttgttaattatctgacctacctccgcctatttcaacgtcctggtgaagacgcgagccgggaaggccgaccgaccaaataacgactattt

Backbone

180 185 190 195 200 205 210 215 220 225 230
Arg Asp Thr Thr Met Pro Val Ala Met Pro Thr Thr Leu Arg Lys Leu Leu Thr Gly Glu Leu Leu Thr Leu Ala Ser Arg Gln Gln Leu Ile Asp Trp Met Glu Ala Asp Lys Val Ala Gly Pro Leu Leu Arg Ser Ala Leu Pro Ala Gly Trp Phe Ile Ala Asp Lys
AmpR

Arg Asp Thr Thr Met Pro Val Ala Met Pro Thr Thr Leu Arg Lys Leu Leu Thr Gly Glu Leu Leu Thr Leu Ala Ser Arg Gln Gln Leu Ile Asp Trp Met Glu Ala Asp Lys Val Ala Gly Pro Leu Leu Arg Ser Ala Leu Pro Ala Gly Trp Phe Ile Ala Asp Lys

Ser Val Val Ile Gly Thr Ala Ile Gly Val Val Asn Arg Leu Ser Asn Val Pro Ser Ser Ser Val Arg Ala Glu Arg Cys Cys Asn Ile Ser Gln Ile Ser Ala Ser Leu Thr Ala Pro Gly Ser Arg Arg Glu Ala Arg Gly Ala Pro Gln Asn Ile Ala Ser Leu

tctggagccggtgagcgtgggtctcgcggtatcattgcagcactggggccagatggtaagccctcccgtatcgtagttatctacacgacggggagtcaggcaactatggatgaacgaaatagacagatcgctgagataggtgcctcactgattaagcattggtaactgtc 5780
agacctcggccactcgcacccagagcgccatagtaacgtcgtgaccccggtctaccattcgggagggcatagcatcaatagatgtgctgcccctcagtccgttgatacctacttgctttatctgtctagcgactctatccacggagtgactaattcgtaaccattgacag

Backbone

235 240 245 250 255 260 265 270 275 280 285
Ser Gly Ala Gly Glu Arg Gly Ser Arg Gly Ile Ile Ala Ala Leu Gly Pro Asp Gly Lys Pro Ser Arg Ile Val Val Ile Tyr Thr Thr Gly Ser Gln Ala Thr Met Asp Glu Arg Asn Arg Gln Ile Ala Glu Ile Gly Ala Ser Leu Ile Lys His Trp *
AmpR

Ser Gly Ala Gly Glu Arg Gly Ser Arg Gly Ile Ile Ala Ala Leu Gly Pro Asp Gly Lys Pro Ser Arg Ile Val Val Ile Tyr Thr Thr Gly Ser Gln Ala Thr Met Asp Glu Arg Asn Arg Gln Ile Ala Glu Ile Gly Ala Ser Leu Ile Lys His Trp *

Asp Pro Ala Pro Ser Arg Pro Asp Arg Pro Ile Met

agaccaagtttactcatatatactttagattgatttaaaacttcatttttaatttaaaaggatctaggtgaagatcctttttgataatctcatgaccaaaatcccttaacgtgagttttcgttccactgagcgtcagaccccgtagaaaagatcaaaggatcttcttgag 5950
tctggttcaaatgagtatatatgaaatctaactaaattttgaagtaaaaattaaattttcctagatccacttctaggaaaaactattagagtactggttttagggaattgcactcaaaagcaaggtgactcgcagtctggggcatcttttctagtttcctagaagaactc

Backbone

ori

atcctttttttctgcgcgtaatctgctgcttgcaaacaaaaaaaccaccgctaccagcggtggtttgtttgccggatcaagagctaccaactctttttccgaaggtaactggcttcagcagagcgcagataccaaatactgtccttctagtgtagccgtagttaggccac 6120
taggaaaaaaagacgcgcattagacgacgaacgtttgtttttttggtggcgatggtcgccaccaaacaaacggcctagttctcgatggttgagaaaaaggcttccattgaccgaagtcgtctcgcgtctatggtttatgacaggaagatcacatcggcatcaatccggtg

Backbone

ori

cacttcaagaactctgtagcaccgcctacatacctcgctctgctaatcctgttaccagtggctgctgccagtggcgataagtcgtgtcttaccgggttggactcaagacgatagttaccggataaggcgcagcggtcgggctgaacggggggttcgtgcacacagcccag 6290
gtgaagttcttgagacatcgtggcggatgtatggagcgagacgattaggacaatggtcaccgacgacggtcaccgctattcagcacagaatggcccaacctgagttctgctatcaatggcctattccgcgtcgccagcccgacttgccccccaagcacgtgtgtcgggtc

Backbone

ori

cttggagcgaacgacctacaccgaactgagatacctacagcgtgagctatgagaaagcgccacgcttcccgaagggagaaaggcggacaggtatccggtaagcggcagggtcggaacaggagagcgcacgagggagcttccagggggaaacgcctggtatctttatagtc 6460
gaacctcgcttgctggatgtggcttgactctatggatgtcgcactcgatactctttcgcggtgcgaagggcttccctctttccgcctgtccataggccattcgccgtcccagccttgtcctctcgcgtgctccctcgaaggtccccctttgcggaccatagaaatatcag

Backbone

ori

```
ctgtcgggtttcgccacctctgacttgagcgtcgatttttgtgatgctcgtcaggggggcggagcctatcgaaaaacgccagcaacgcggcctttttacggttcctggccttttgctggccttttgctcacatgttctttcctgcgttatcccctgattctgtggataac
                                                                                                                                                                              6630
gacagcccaaagcggtggagactgaactcgcagctaaaaacactacgagcagtccccccgcctcggatagctttttgcggtcgttgcgccggaaaaatgccaaggaccggaaaacgaccggaaaacgagtgtacaagaaaggacgcaataggggactaagacacctattg
```

Backbone

ori

```
cgtattaccgcctttgagtgagctgataccgctcgccgcagccgaacgaccgagcgcagcgagtcagtgagcgaggaagcggaagagcgcccaatacgcaaaccgcctctccccgcgcgttggccgattcattaatgcagctggcacgacaggtttcccgactggaaagc
                                                                                                                                                                              6800
gcataatggcggaaactcactcgactatggcgagcggcgtcggcttgctggctcgcgtcgctcagtcactcgctccttcgccttctcgcgggttatgcgtttggcggagaggggcgcgcaaccggctaagtaattacgtcgaccgtgctgtccaaagggctgacctttcg
```

Backbone

```
gggcagtgagcgcaacgcaattaatgtgagttagctcactcattaggcaccccaggctttacactttatgcttccggctcgtatgttgtgtggaattgtgagcggataacaatttcacacaggaaacagctatgaccatgattacgccaagctatttaggtgacactata
                                                                                                                                                                              6970
cccgtcactcgcgttgcgttaattacactcaatcgagtgagtaatccgtggggtccgaaatgtgaaatacgaaggccgagcatacaacacaccttaacactcgcctattgttaaagtgtgtcctttgtcgatactggtactaatgcggttcgataaatccactgtgatat
```

Backbone

CAP binding site | -35 | -10 | lac promoter | lac operator | M13 rev | SP6 promoter

```
gaatactc   3′
      ••• 6978
cttatgag   5′
```

Backbone

SP6 promoter
